# Dissociating and linking divergent effects of emotion on cognition: insights from current research and emerging directions

**DOI:** 10.3389/fpsyg.2025.1483373

**Published:** 2025-05-14

**Authors:** Florin Dolcos, Ekaterina Denkova, Alexandru D. Iordan, Andrea T. Shafer, Guillén Fernández, Sanda Dolcos

**Affiliations:** ^1^Department of Psychology, University of Illinois, Urbana-Champaign, IL, United States; ^2^Neuroscience Program, University of Illinois, Urbana-Champaign, IL, United States; ^3^Beckman Institute for Advanced Science and Technology, University of Illinois, Urbana-Champaign, IL, United States; ^4^Department of Psychology, University of Miami, Coral Gables, FL, United States; ^5^Department of Psychology, University of Michigan, Ann Arbor, MI, United States; ^6^Centre for Neuroscience, University of Alberta, Edmonton, AB, Canada; ^7^Donders Institute for Brain, Cognition, and Behaviour, Radboud University Medical Center, Nijmegen, Netherlands

**Keywords:** attention, emotional memory, working memory, emotional distraction, emotion perception, stress, cognitive aging, affective disorders

## Abstract

This century has witnessed unprecedented increasing interest in the investigation of emotion-cognition interactions and the associated neural mechanisms. The present review emphasizes the need to consider the various factors that can influence enhancing and impairing effects of emotion on cognition, in studies of both healthy and clinical groups. First, we discuss advances in understanding the circumstances in which emotion enhances or impairs cognition at different levels, both *within the same processes* (e.g., perception, episodic memory) and *across different processes* (i.e., episodic vs. working memory). Then, we discuss evidence regarding these opposing effects of emotion in a larger context, of the *response to stressors,* and linked to the role of individual differences (personality, genetic) affecting stress sensitivity. Finally, we also discuss evidence linking these opposing effects of emotion in a *clinical group* (PTSD), where they are both deleterious, and based on comparisons *across groups* with opposing affective biases: healthy aging (*positive bias*) vs. depression (*negative bias*). These issues have relevance for understanding mechanisms of emotion-cognition interactions in healthy functioning and in psychopathology, which can inspire training interventions to increase resilience and well-being.

## 1 Introduction

**Figure 1:**
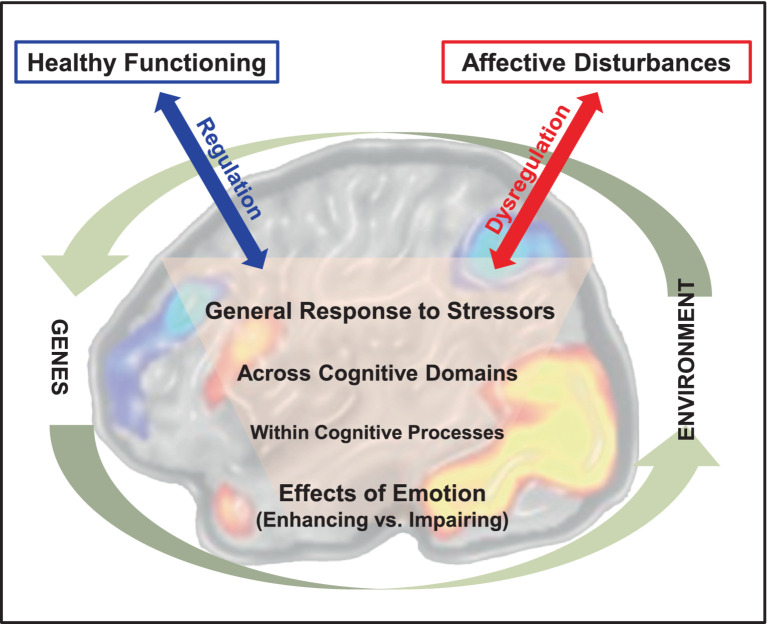
Emotion-cognition interactions in the brain and their relation to adaptive and maladaptive outcomes. The diagram illustrates opposing effects of emotion on cognition at increasing levels of complexity in emotion-cognition interactions. The involvement of brain mechanisms at all these levels is suggested by the background brain image depicting activations in brain regions that are part of two main neural systems: a dorsal neural system involved in “cold” cognitive/executive processing (illustrated by cold-colored brain activations) and a ventral system involved in “hot” emotion processing (illustrated by warm-colored brain activations). The effective vs. dysfunctional engagement of regulatory mechanisms in emotion-cognition interactions are depicted by the blue and red arrows, linked to adaptive vs. maladaptive outcomes, respectively. Finally, these interactions occur in the larger context circumscribed by interplays between genetic and environmental factors influencing them. It should be noted that the latter interplays are indirect, as genes do not affect directly our environment and the environment does not actually affect our genetic code. Instead, the genes making up the genetic code (genotype) are expressed in phenotypes that affect the environment, which in turn affects transcription and gene expression (epigenetics). The brain image was adapted from Dolcos and McCarthy (2006), with permission.

Emotion can enhance or hinder various aspects of our cognition and behavior. For instance, the emotional charge of an event can increase attention to and memory for that event, leading to enhanced memory, whereas task-irrelevant emotional information may lead to increased distraction and hence can impair cognitive performance. The overarching goal of this review is to discuss evidence regarding factors that influence opposing effects of emotion on cognitive processing at different levels (Figure 1), and the associated neural mechanisms, and to highlight the need to consider such factors in studies investigating emotion-cognition interactions in healthy and clinical groups. These issues have relevance for understanding mechanisms of emotion-cognition interactions in healthy functioning and in emotional disturbances, where such opposing effects^1^ of emotion tend to be exacerbated and deleterious. Notably, we do not aim to present the available evidence regarding the impact of emotion on different aspects of cognition as part of a coherent theoretical framework. The main rationale for our approach is to increase awareness of the fact that such effects can occur and be identified at different levels. This is because the tendency is to be treated in isolation, in separate literatures (e.g., attention, perception, memory). Hence, the present review provides a more comprehensive image of these divergent effects and of their possible links.

The basic idea that emotion can have divergent effects on different cognitive aspects is not completely novel. Instead, what is novel is identification and consideration of such effects at different levels. Our first attempt to increase awareness about this goes back more than 10 years ago, when we organized a Frontiers Special Issue/Research Topic (the first in the *Emotion Science* section) tackling for the first time this matter in a comprehensive way. Our initiative was very well received and has resulted in a collection of 60+ manuscripts, received from a large number of outstanding contributors (200+, in total), pointing to divergent patters in a variety of aspects. Summarized in an Editorial and compiled in an edited book (Dolcos et al., 2015, respectively), our special issue has been at the top of popularity among the Frontiers Research Topics. Importantly, by all accounts, our initiative was very successful in increasing awareness of such patterns in the impact of emotion on cognition.

The efforts to increase awareness have contributed to further clarification of the circumstances in which emotion enhances or impairs cognition and prepared the ground for further theoretical advancements. A concrete example, which we are also highlighting here, is the recent reconciliation of evidence regarding opposing effects of emotion on relational memory (Bogdan et al., 2024). Initially, a pattern was emerging in the emotional memory literature, whereby the enhancing effects of emotion were not systematically observed in all aspects of memory (e.g., central vs. peripheral; Kensinger, 2009). Then, more recently, evidence pointed to opposing effects of emotion on item (what) vs. relational memory (item-context associations), whereby emotion enhanced item memory but impaired memory for item-context associations (Bisby and Burgess, 2017). However, we recently provided further evidence regarding the circumstances in which emotion enhances or impairs relational memory, and proposed a new theoretical account (Bogdan et al., 2024). It should be noted that, although there are various accounts proposing to explain the impact of emotion on episodic memory, no single theory covers all aspects of emotion-memory interactions. In section 2.2.4, we illustrate the difficulty in reaching a comprehensive theoretical account, even within the same domain (Figure 4), which makes it even more difficult identification of a coherent theoretical framework that covers all levels of emotion-cognition interactions. Hence the present goal of increasing awareness that such divergent patterns can be identified at different levels, while also pointing to emerging theoretical accounts resulted from research aimed at further understanding these divergent patterns in specific domains (see Figure 6, which introduces a new model of emotion-memory interactions).

**Figure 2:**
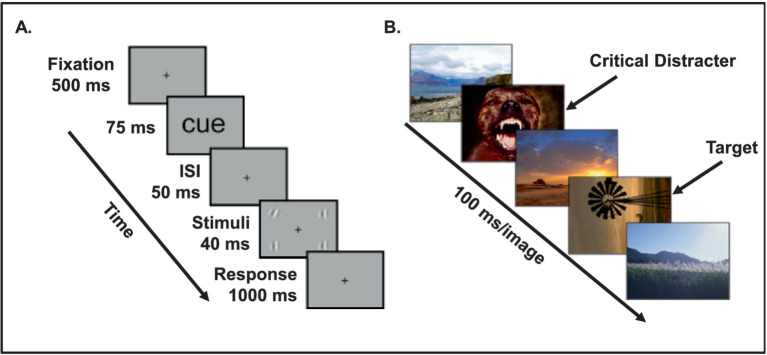
Experimental paradigms used to investigate the link between the timing of emotion processing and opposing effects of emotion on visual perception. **(A)** A briefly presented emotional cue (e.g., fearful face) enhances visual processing of neutral targets (e.g., low-contrast gratings) following a short interval (50 ms), resulting in reduced threshold of detecting the orientation of the neutral targets. From Phelps et al. (2006), with permission. **(B)** Emotional distracters impair visual processing of neutral target following a longer interval (100 ms), thus resulting in impaired ability to identify a neutral target. Notably, this impairment progressively diminishes as the time interval between the emotional stimulus and the neutral target is increased and, ultimately, identification of a neutral target is again enhanced by emotion. From Ciesielski et al. (2010), with permission.

**Figure 3:**
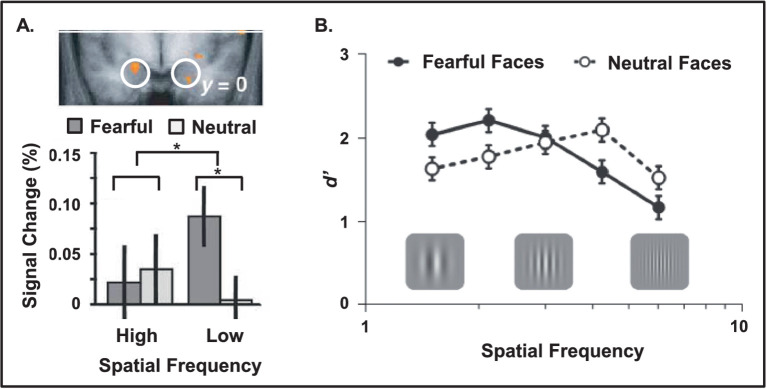
Increased amygdala sensitivity and enhanced behavioral performance linked to low spatial frequency emotional stimuli. **(A)** Amygdala shows increased sensitivity for low compared to high spatial frequency emotional information (e.g., fearful faces displayed with different frequency filters). The top view displays the bottom part of a coronal section of the brain, at the level of the amygdala (white circles). Red areas identify regions showing a significant emotional expression (fearful vs. neutral) x spatial frequency (high vs. low) interaction, evident in the bar graph. Adapted from Vuilleumier et al. (2003), with permission. **(B)** Opposing effects of emotional cues linked to spatial frequency of the targets. Fearful cues enhanced detection of low and impaired detection of high spatial frequency targets following after a short interval (40 ms). From Bocanegra and Zeelenberg (2009b), with permission.

**Figure 4:**
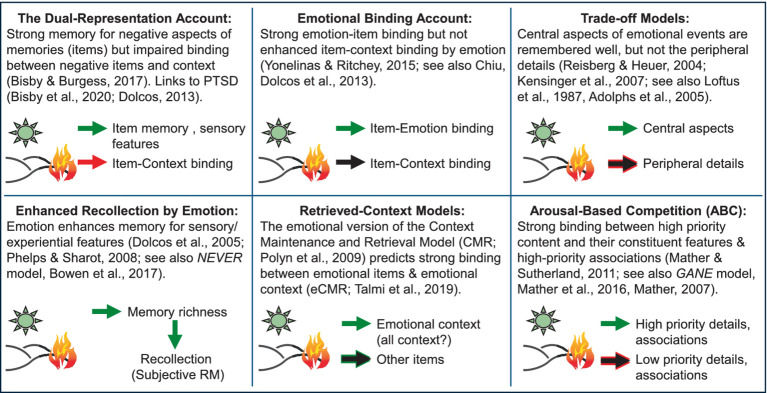
Disagreement among models of relational memory, emphasizing the need for unifying theoretical accounts. Figure developed in collaboration with Deborah Talmi, Daniela Palombo, and Mathias Weymar for a symposium at the Cognitive Neuroscience Society Annual Meeting (Dolcos and Talmi, 2024). PTSD, Post-traumatic stress disorder; NEVER, Negative Emotional Valence Enhances Recapitulation; GANE, Glutamate Amplifies Noradrenergic Effects. The green arrows indicate enhancing effects of emotion on memory; the red arrows indicate impairing effects of emotion on memory; the black arrows indicate no effects of emotion on memory (neither enhancing not impairing).

**Figure 5:**
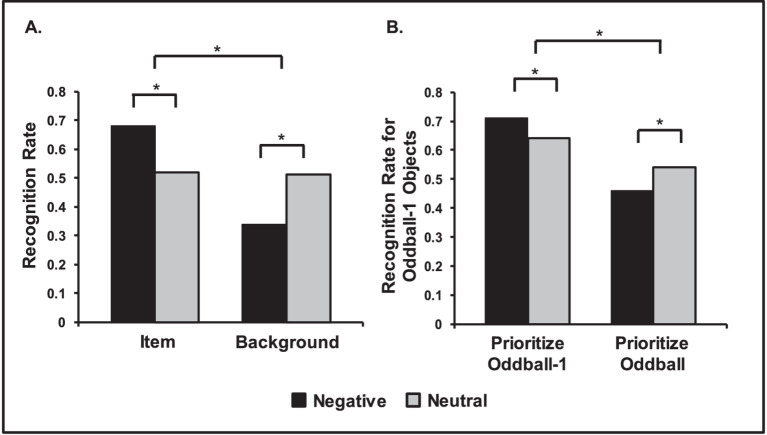
Opposing effects of emotion on episodic memory. **(A)** Central vs. Peripheral memory trade-off for items and background pictures. Negative items were remembered better than neutral items, but memory for backgrounds was lower when presented with negative items than when presented with neutral ones. Adapted from Waring et al. (2010), with permission. **(B)** Opposing effects of emotion on memory linked to prioritization. Neutral objects were better remembered when followed by negative compared to neutral oddball images, if participants prioritized the neutral objects (Prioritize Oddball-1 Condition). However, memory was worse when subjects prioritized the oddballs, instead (Prioritize Oddball Condition). Adapted from Sakaki et al. (2014a), with permission. *Significant differences.

**Figure 6:**
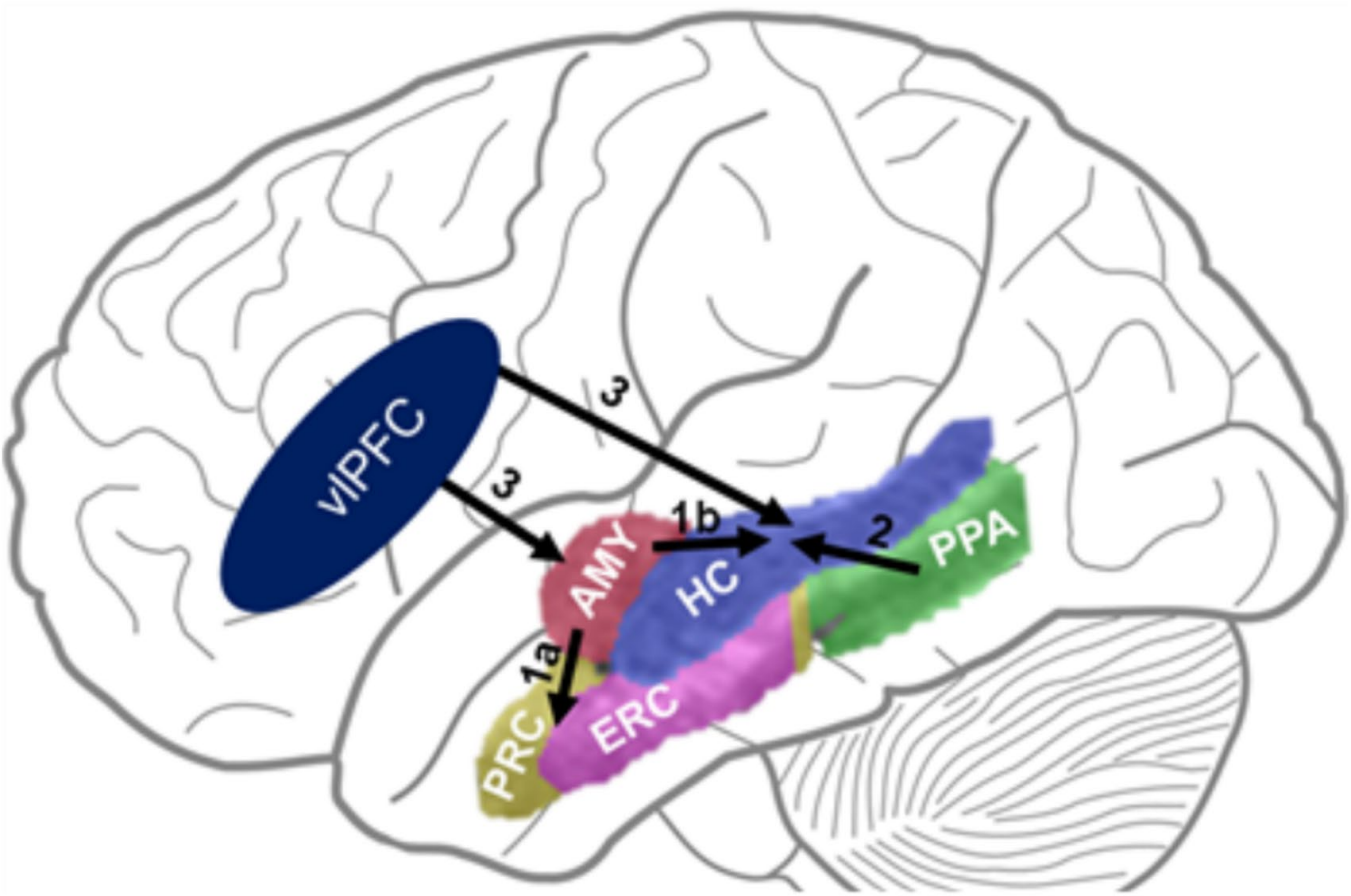
The DEAME model. Paths 1 & 2 show bottom-up influences from emotion- (AMY) and perception-related (PPA) areas on memory-related MTL regions. Path 3 shows top-down PFC influences on activity in MTL regions. Challenging the view positing agonistic (1a) vs. antagonistic (1b) influences of AMY on brain regions involved in item (PRC) vs. relational (HC) memory, respectively, we show that both paths (1a & 1b) have synergistic engagement leading to enhanced RM, together with path 2. Also, paths 1 & 2 are both susceptible to influences from the left vlPFC. AMY, Amygdala; HC, Hippocampus; PRC, Perirhinal Cortex; ERC, Entorhinal Cortex; PPA, Parahippocampal Place Area (part of the Parahippocampal Cortex Proper); PFC, vlPFC, Ventrolateral Prefrontal Cortex.

Ten years after the conclusion of our special issue focusing on these aspects (Dolcos et al., 2015), many topics are still current. Below, we will briefly introduce and then discuss them in detail. First, enhancing and impairing effects of emotion can be identified *within the same cognitive processes/domains*, such as perception and episodic memory (i.e., memory for specific personal events). Opposing effects of emotion in perception can be identified linked to the context in which emotional information is processed (goal-relevant or irrelevant) (Ohman et al., 2001a; Ohman et al., 2001b), linked to the timing of its processing (simultaneous or asynchronous) (Bocanegra and Zeelenberg, 2009a, 2011b; Ciesielski et al., 2010; McHugo et al., 2013; Ohman et al., 2001a; Phelps et al., 2006), and linked to the spatial frequency of visual information (high or low spatial frequency) (Bocanegra and Zeelenberg, 2009b, 2011a; Vuilleumier et al., 2003). Regarding episodic memory, opposing effects of emotion can be attributed to different accounts, including central vs. peripheral effects (Kensinger, 2009) and high vs. low prioritization of information (Mather and Sutherland, 2011). Moreover, an important topic of research in this area concerns opposing effects of emotion on associative or relational memory (Chiu et al., 2013), which may be differentially affected in both healthy functioning and clinical condition, including neurological (Alzheimer’s), affective (mood and anxiety disorders), and other disturbances (schizophrenia).

Second, there is also emerging evidence of opposing effects of emotion *across cognitive processes/domains*, which also emphasizes the link and dissociation between immediate and long-term effects of emotional distraction on perception and working memory (Dolcos, 2013; Shafer and Dolcos, 2012), on the one hand, and episodic memory, on the other hand. For instance, task-irrelevant emotional information can impair ongoing cognitive processing, while also enhancing long-term memory for the distracters themselves. Seeing the scene of a tragic accident while driving may temporarily distract us from the main task (driving), while also leading to better memory for the distracting information (the totaled cars). Novel brain imaging evidence regarding these phenomena points to both overlapping and dissociable neural mechanisms mediating these opposing effects of emotion (Dolcos et al., 2013; Shafer and Dolcos, 2012), and highlights the role of other factors, such as the load of the main cognitive task (Shafer and Dolcos, 2012; Shafer et al., 2012).

Third, in a *larger context of the stress response,* emotional stressors can lead to opposing effects depending on the context and degree. Optimal levels of stress may temporarily increase cognitive performance (e.g., nervousness about an upcoming important exam may motivate us to study harder), whereas high levels of stress can impair performance (e.g., overwhelming worry in the anticipation of, or during, a difficult exam may impair our ability to stay focused and perform optimally) (Diamond et al., 2007). Moreover, chronic and/or extreme levels of stress can lead to clinical conditions (Arnsten, 2009; Roozendaal et al., 2009), such as post-traumatic stress disorder (PTSD), which are associated with longer-lasting cognitive impairments. An interesting emerging finding in this area points to the role of subjective or objective control upon stressful situations (Henderson et al., 2012; Kerr et al., 2012; Mereu and Lleras, 2013), in determining the beneficial or detrimental impact on cognitive processing. In addition, recent research is also considering the role of individual differences in response to stressors, which can lead to adaptive or *mal*adaptive consequences. Thus, it is important to consider both factors related to the stressors themselves and factors related to variations (personality, genetic) in the individuals’ responses to stressful situations.

Fourth, the co-occurrence of enhancing and impairing effects of emotion is probably most evident in *affective disturbances*, such as PTSD, which are characterized by increased sensitivity to emotional distraction and impaired cognitive control (Hayes et al., 2012). Thus, both of these opposing effects of emotion are exacerbated and deleterious. For example, uncontrolled recollection of traumatic memories in PTSD may interfere with ongoing cognitive processing. Evidence from PTSD studies points to altered interactions between the mechanisms that are typically responsible for enhancing vs. impairing effects of emotion in healthy functioning (Dolcos, 2013). Specifically, as discussed in Section 5, there is evidence suggesting that non-specific responses to cues for trauma-related memories, presented as task-irrelevant distraction (Morey et al., 2009), may reflect non-specific initial encoding of decontextualized memories for the traumatic events due to heightened arousal (Hayes et al., 2011).

Finally, there is also intriguing converging evidence from *across-fields comparisons* of findings from groups with opposing emotional biases, such as healthy aging (showing a positive bias, Mather, 2012; Mather and Carstensen, 2005) vs. depression (showing a negative bias). Interestingly, these opposing biases are linked to opposite effects on the ability to control emotions in these groups – enhanced emotion regulation in healthy aging (Mather, 2012; St Jacques et al., 2010; Dolcos et al., 2014) vs. impaired emotion control or emotion *dys*regulation in depression (Mayberg, 1997). Thus, direct comparisons of these groups with opposing emotional biases and emotion regulation abilities, along with studies aiming at elucidating the mechanisms of enhanced emotional resilience in healthy aging, provide an exciting possible research avenue to address mental health issues. All these issues will be discussed in detail in the next sections. The review ends with concluding remarks and a discussion of open issues and future directions.

## 2 Opposing effects of emotion within the same cognitive domain

The findings discussed in this and the next section are based mainly on manipulations of transient emotional responses, which typically elicit phasic influences on cognitive processing, and we only briefly reference tonic effects of longer-lasting emotional states, such as mood and stress. Complementing this body of evidence, Section 4 specifically focuses on the impact of stress on cognitive processing. Of note, emotional reactions and states are separable phenomena, with the former being relatively more intense and short in duration and the latter being relatively more diffuse and prolonged, and they may exert different influences on cognition and behavior (Olsson and Öhman, 2009; Rottenberg and Gross, 2003; Watson, 2000).

### 2.1 Opposing effects of emotion on visual perception and attention

Investigation of the impact of emotion on visual perception and attention has shown that visual processing of affective information is prioritized over non-affective information. Evidence for this prioritization is provided by research using detection, visual search, attentional capture, and attentional blink paradigms. Human and non-human primate investigations of emotion processing have provided evidence that the impact of emotion on visual perception and attention is largely linked to the amygdala (AMY) (Anderson and Phelps, 2001; Lim et al., 2009; Phelps, 2006). While the routes by which AMY influences processing in sensory cortices to alter stimulus processing in the human brain remain debated (see Pessoa, 2013, for a review), both human lesion and neuroimaging data show that this brain region plays a pivotal role in low-level perceptual and attentional modulations by emotion (Anderson and Phelps, 2001; Lim et al., 2009). Evidence from studies investigating the effect of prioritization of emotion processing show that emotion can both impair and enhance performance, but the directionality of these effects depends on a number of factors. Below we will discuss evidence regarding the role of the following three aspects in determining enhancing or impairing effects of emotion on visual perception and attention: (1) the *context* of emotion processing (*task-relevant vs. irrelevant*), (2) the *timing* of emotion processing (*simultaneous vs. asynchronous*), and (3) the spatial frequency of visual emotional information (*low vs. high spatial frequency*).

#### 2.1.1 Context of emotion processing (task-relevant vs. task-irrelevant)

An important factor in determining the impact of emotion on perception and attention is whether emotional stimuli serve as targets (task-relevant) or distracters (task-irrelevant). Rapid serial visual presentation paradigms (RSVP) and the attentional blink phenomenon (Dux and Marois, 2009; Raymond et al., 1992) offer good examples of how altering the context of emotion processing results in a different impact of emotion on behavior. In RSVP studies, streams of stimuli (words or pictures) are presented in a rapid succession, with individual stimuli presented one at a time, typically displayed for 80–125 ms each and with no interstimulus interval (ISI). In such paradigms, a so-called *attentional blink* occurs when the processing of an initial target stimulus (T1) presented in the stimulus stream impairs the ability to detect another target stimulus (T2) that is presented soon after the first target stimulus. Interestingly, when T1 is emotional, and no report of T1 is required, T1 becomes a “distracter” stimulus, and the time interval during which the ability to detect T2 becomes longer (i.e., the “blink”) (McHugo et al., 2013). This is also referred to as “emotion-induced blindness” (Most et al., 2005). However, when T2 is emotional, the ability to accurately detect T2 is enhanced and the duration of the “blink” produced by processing T1 is reduced (Keil and Ihssen, 2004).

This example emphasizes a generalization that can be made about the effect of emotion on perception and attention. When an exogenous emotional stimulus is task-relevant, the prioritization of processing for affective information results in task-enhancement, whereas when task-irrelevant, the boost in processing resources received by the now distracting emotional stimulus depletes the resources available for initial or continued processing of a target stimulus. These opposing effects observed behaviorally seem to be linked to the same neural mechanisms that allow increased mobilization and allocation of processing resources associated with the prioritization of affective information and involve AMY.

#### 2.1.2 The timing of emotion processing (simultaneous vs. asynchronous)

A related factor that influences the effects of emotion on perception and attention is the timing of presenting emotional and non-emotional stimuli. There are predominantly two main ways in which tasks are designed to examine the impact of emotion on perception and attention. In one approach, emotional stimuli are (i) presented *simultaneously* with other stimuli, whereas in the other emotional and non-emotional stimuli are presented *asynchronously* and are either (ii) distributed evenly across the screen or are (iii) limited to specific screen locations. Each of these approaches can result in either an impairing or enhancing effect of emotion on perception and attention, in relationship to the factor described above – i.e., whether the emotional information is task-relevant or not.

An example of the first approach (i) is the pop-out visual search, where emotional stimuli serve as either targets (task-relevant) or distracters (task-irrelevant). In pop-out visual search, a number of items are displayed at the same time, with all items, but one, identical. The non-identical item differs from the identical items to a degree that makes it easily identifiable and is, therefore, said to “pop-out” of the display. As highlighted above, when an emotional item is presented as target, the time required to detect it is reduced. Alternatively, when emotion is presented as distraction, the time required to detect a non-emotional target is impaired (Ohman et al., 2001b). Therefore, when emotion is presented simultaneously with non-emotional items, the direction of emotion’s impact will also be dependent on whether the emotional stimulus is the target (task-relevant) or serves as distraction (task-irrelevant).

Converging findings from investigations that incorporate asynchronous presentation of emotional and non-emotional items shows that differences in the stimulus duration and the length of the interstimulus interval (ISI) determine whether task-irrelevant emotional information enhances or impairs performance of an asynchronously presented non-emotional target. For example, in the case of (ii), briefly presented and distributed fear stimuli with a short ISI between the fear stimulus and a non-emotional target stimulus facilitate the perceptual processing of the non-emotional target and therefore enhance performance (Bocanegra and Zeelenberg, 2009b, 2011a; Phelps et al., 2006). Moreover, in the case of (iii), if fear stimuli also serve as spatial cues for the location of subsequent non-emotional targets, the affective and attentional information of the cue interact to boost perceptual processing of the non-emotional target even further (Figure 2A) (Phelps et al., 2006).

Interactions between the timing of presenting emotional stimuli and manipulations of their task-relevance can also be influenced by variations in the duration of the interval between an emotional stimulus and a target stimulus. For instance, in the context of the findings regarding the attentional blink discussed above, if the stimulus duration for an emotional item and the interval between an emotional item and a target item are longer, then the perceptual processing is impaired (Figure 2B) (Ciesielski et al., 2010; McHugo et al., 2013). However, if the interval is extended even further (i.e., after the attentional blink period), then emotion again shows an enhancing effect on target detection (Bocanegra and Zeelenberg, 2009a; Ciesielski et al., 2010). Consequently, when emotion is task-irrelevant but presented briefly and immediately prior to a non-emotional target, the non-emotional target receives a boost in processing and performance is enhanced. However, if more in-depth processing of task-irrelevant processing is allowed to occur, as a result of longer stimulus durations, and the target has as larger temporal gap separating it from the task-irrelevant emotion, the processing resources available to detect the non-emotional target are depleted and hence performance is impaired. Importantly, however, this impairment is only momentary and non-emotional targets presented immediately after the “blink” period also receive a boost in processing resources and performance is enhanced.

The differential impact of emotion depending on the temporal delay between emotional stimuli and non-emotional targets may, at least in-part, be explained by the time course of emotion-attention interactions on visual processing, and also involves AMY. For instance, investigations exploring the temporal aspects of emotion and attention on visual processing of task-irrelevant emotional information in the AMY, under different conditions of attentional demand in the main task (low vs. high demand), have shown differences in the susceptibility of the AMY response to affective and attentional information, over the time course of the response epoch (Luo et al., 2010; Pourtois et al., 2010). Specifically, the initial modulation occurring early in the time course was due to emotion and was invariant to the attention demands of the main task. In contrast, the later modulation was sensitive to the interaction between the emotion and attentional demands, such that an emotion response was found only when the task demands were low (Luo et al., 2010) or the emotional stimuli were task-relevant (Pourtois et al., 2010).

Regarding the neural mechanisms, the enhancement in target detection immediately following an emotional stimulus (Bocanegra and Zeelenberg, 2009b, 2011a, 2011b; Phelps et al., 2006) could result from an early, attention invariant, emotional response in the AMY, whereas the subsequent impairment in target detection could result from the later dampening of the AMY’s response to emotion by concurrent attentional demands, coupled with the possibility that continued higher-ordered processing of the emotional stimulus diminishes the resources available for later perceptual processing of a target stimulus. The two different time windows in the AMY response modulation by emotion and attention map onto the first two time windows of target presentation, where opposing behavioral effects are identified (i.e., immediate enhancement and subsequently impaired). However, it is unclear how AMY activity relates to the later stage enhancement in target detection. One possibility is that this later enhancement is due to the release of resources that were “consumed” by the emotional relative to neutral processing. In this regard, it is also possible that, when “releasing” the emotional stimulus, the system does not gradually reset to baseline where there is a balance between bottom-up and top-down processing, but slightly oversets toward a ready-state for bottom-up processing.

Finally, an often ignored but important aspect related to such emotion-cognition interplay is the timing of neurochemical modulations associated with these phenomena, which are most evident in investigations manipulating the stress response.^2^ Indeed, converging evidence from animal and human studies (reviewed in Hermans et al., 2014) points to multiple waves of neurochemical events, such as catecholamine and corticosteroid release, that influence widely distributed neuronal populations and may have opposing effects at different time scales. For instance, animal studies have demonstrated an association between prompt increases of central catecholamine levels (e.g., norepinephrine and dopamine) and enhanced scanning of the environment, following exposure to a stressor (Aston-Jones and Cohen, 2005), whereas, in humans, stress induction has been associated with enhanced detection of the second target in the attentional blink paradigm (Schwabe and Wolf, 2010). Such phenomena have been linked to a shift in locus coeruleus activity (Sara and Bouret, 2012), the main supplier of central norepinephrine, which may in turn exert opposing influences on AMY (enhancement) and the prefrontal cortex (impairment), via adrenoreceptors (Birnbaum et al., 1999; Wang et al., 2007). On the other hand, corticosteroids potentiate short-term catecholamine release under stress and also exert slow genomic effects (>1 h after stressor exposure), by altering gene transcription (Joels et al., 2012). This effect has been linked to downregulation of AMY response (Henckens et al., 2010) and enhanced response in the prefrontal cortex (PFC), coupled with improved cognitive performance (Henckens et al., 2011). Thus, future research needs to carefully dissociate between influences of emotion on cognitive processing at different time scales and better control for such temporal factors.

#### 2.1.3 The spatial frequency of emotional information (low vs. high spatial frequency)

Another important factor in determining the impact of emotion on processing visual information is the spatial frequency of the stimuli. Simply put, spatial frequency is a measure of the density of visual information in a fixed area of space. Less dense or coarse space has low spatial frequency, whereas more dense or fine-grained space has high spatial frequency. The visual system is organized to differently accommodate these two types of visual information. Magnocellular cells and pathways are tuned to respond to low spatial frequency information, and parvocellular cells and pathways are tuned to high spatial frequency information. Investigations of amygdalar anatomy in non-human primate show that there is a predominance of magnocellular efferent projections from AMY to the visual cortices, suggesting a bias in the type of information that is enhanced (Amaral et al., 2003). Consistent with this idea, investigation of human AMY response to low vs. high spatial frequencies showed that AMY is more sensitive to low spatial frequencies (Figure 3A) (Vuilleumier et al., 2003). Therefore, it may be the case that the initial boost in perceptual processing of emotional information and/or non-emotional targets that immediately precede an emotional stimulus is found only for low spatial frequency information, while high spatial frequency information is impaired. A study examining this idea found that a fearful cue enhanced the ability to accurately identify a low spatial frequency target, but impaired accuracy for a high spatial frequency target (Figure 3B) (Bocanegra and Zeelenberg, 2009b).

When considering the function of a quick detection system to identify potential threat, from a survival perspective, it is more beneficial to first determine the presence of a potential threat, rather than the exact nature of the threat. Hence, in this sense, the “*what is it*” question matters once quick action is taken based on the initial detection, and being at a safe distance will then allow for continued processing. Indeed, the visual system is designed for a quick detection of threat with magnocellular neurons, responding faster and being linked to peripheral vision, and with parvocellular neurons, responding slower and being linked to foveal vision (Maunsell et al., 1999). As a result, a fine-grained distinction of a potential threat will only occur after fixation which is subsequent to initial detection. Along this line, and based on inherent trade-offs across these two pathways (i.e., peripheral vs. foveal concentration, fast vs. slow response, crude vs. fine-grained information), a boost in magnocellular-based visual processing should also increase temporal resolution, while a boost in parvocellular-based visual processing should impair temporal resolution. This idea was investigated using a temporal gap detection task (Bocanegra and Zeelenberg, 2011a). In this task, the low spatial frequency information in a distributed emotional cue was found to enhance the detection of a temporal gap in the presentation of a target stimulus, relative to a neutral cue. Moreover, this study also differentiated the effects of low spatial frequency emotional information on temporal vs. spatial resolution. While temporal resolution was enhanced, the low spatial frequency information of a distributed emotional cue impaired the ability to detect high spatial resolution differences in target stimuli.

Overall, the evidence reviewed in this section shows that the opposing effects of emotion on visual perception and attention have been identified in terms of dissociations between task-relevant and task-irrelevant emotional stimuli, simultaneous vs. asynchronous presentation of stimuli, and low vs. high spatial frequency information. These factors may be considered either independently or as interacting with one another, and future research should consider these factors and their possible interactions in predicting and interpreting findings regarding opposing effects of emotion on visual processing.^3^

### 2.2 Opposing effects of emotion on episodic memory

There is strong evidence from both animal and human research that emotional events are overall better remembered than neutral events (Dolcos and Denkova, 2008; Dolcos et al., 2012; Dolcos et al., 2006; McGaugh, 2005; Phelps, 2004). The effects of emotion on episodic memory in humans have been typically investigated using experimenter-generated stimuli, such as lists of words or sets of pictures, varying in their emotional content, which participants are encoding in laboratory settings and then their memory is tested at different intervals (e.g., from minutes to several months). Such investigations have provided strong evidence that enhanced memory for emotional stimuli is linked to amygdala’s involvement and its interaction with memory-related medial temporal lobe (MTL) regions (hippocampus and the associated entorhinal, perirhinal and parahippocampal cortices). In addition, the memory-enhancing effect of emotion can also benefit from the engagement of higher order cognitive brain regions (e.g., the prefrontal and parietal cortices), through their involvement in semantic, working memory, and attentional processing (Dolcos and Denkova, 2008; Dolcos et al., 2012; Dolcos et al., 2017a).

However, there is also evidence that not all aspects of an event benefit from such enhancement by emotion (Kensinger, 2009). Whereas emotion enhances memory formation for isolated or intrinsic properties of emotional items, it can also impair memory for other extrinsic aspects or memory for items in relation to other items (relational or associative memory) (Kensinger, 2009; Mather, 2007). Some evidence suggests that these opposing effects of emotion^4^ are due to *central vs. peripheral trade-offs* (Kensinger, 2009), and other studies emphasize the *level of priority* (high vs. low) of emotional information in understanding enhancing vs. impairing effects of emotion on memory (Mather and Sutherland, 2011). Additionally, it has also been proposed that the opposing effects of emotion on memory might also depend on the *type of associations* (Chiu et al., 2013). Below, we discuss evidence regarding these three aspects, as well as novel evidence reconciling the opposing effects of emotion on item vs. relational memory (Bogdan et al., 2024). The latter findings also point to possible training interventions to reduce unwanted attentional biases and increase memory specificity and well-being (e.g., in affective disorders and aging).

Notably, despite various attempts, there is no unifying theory that accounts for all behavioral patterns regarding the impact of emotion on various aspect of episodic memory (see Figure 4). Although some models can account for more of the available evidence than others, no single theoretical account can explain the variety of findings. Although the amygdala has a central role in modulating emotional memories in all models, its engagement is not instrumental in the same way. For instance, a prominent view suggests that impaired relational memory by emotion is due to inhibitory/antagonistic effects exerted by the amygdala on hippocampal activity (Bisby and Burgess, 2017). However, challenging this view, as discussed below, we propose a new model (Bogdan et al., 2024) positing that emotion enhances relational memory through synergistic/agonistic engagement of the amygdala and hippocampus (see Figure 6). We expect that the findings by Bogdan et al. (2024) will fuel future research aimed at further clarifying the circumstances in which emotion enhances or impairs episodic relational memory.

#### 2.2.1 The central vs. peripheral trade-off in the impact of emotion on memory

The observation that emotion enhances memory for central aspects and impairs memory for peripheral details has been initially reported in the eyewitness memory literature, which has coined the term “*weapon focus effect*.” This refers to the tendency in crime witnesses to focus on the weapon and miss other details of the event (Christianson, 1992; Loftus et al., 1987). More recent research of emotional memory has referred to this phenomenon as the *central vs. peripheral trade-off* (Kensinger, 2009), the *narrowing effect of emotion on memory* (Reisberg and Heuer, 2007), or as *tunnel memory* (Safer et al., 1998). For example, Kensinger et al. (2007a) suggests a trade-off effect in memory, in which central aspects of stimuli are better remembered at the expense of remembering peripheral details (for reviews, see Kensinger, 2009; Steinmetz and Kensinger, 2013). Thus, the trade-off refers to increased memory for emotional vs. neutral items, and decreased memory for backgrounds associated with emotional vs. neutral items (see Figure 5A).

This effect is typically investigated by presenting emotionally aversive or neutral objects against neutral backgrounds (e.g., an alligator by a river, and a squirrel in a forest). Such investigations showed better memory for emotional than for neutral objects, but worse memory for neutral backgrounds when paired with emotional objects than when paired with neutral objects (Kensinger et al., 2007b; Mickley Steinmetz et al., 2012; Waring and Kensinger, 2009; Waring et al., 2010). Brain imaging studies investigating the neural correlates of these effects have shown that AMY is involved in memory-enhancing effects for aspects that are intrinsically linked to the emotional items themselves, but not for other aspects, such as the context/background in which they are encoded (Dougal et al., 2007; Kensinger et al., 2007a; Kensinger and Schacter, 2006).

#### 2.2.2 The role of prioritization in the impact of emotion on memory

Complementary evidence suggests that opposing effects of emotion on memory are related to prioritization processes, as emphasized by the *ABC (Arousal-Biased Competition) Theory* (Mather and Sutherland, 2011). According to this theory, emotional arousal enhances encoding of high priority^5^ information at the expense of low priority information (Mather and Sutherland, 2011). In a series of studies investigating the effects of emotional arousal as a function of prioritization, Mather et al. showed that emotional stimuli can enhance learning for preceding prioritized neutral objects, but impairs memory for preceding non-prioritized objects (Figure 5B) (Lee et al., 2012; Lee et al., 2014; Sakaki et al., 2014a; Sutherland and Mather, 2012). Neural evidence points to dissociable AMY involvement according to whether information is prioritized or not, as suggested by recent brain imaging studies identifying greater coupling between AMY and perceptual areas for processing high-priority stimuli (Lee et al., 2014). The ABC model can also be linked to accounts considering motivational factors to clarify the impact of emotion on memory (Levine and Edelstein, 2009; Sander et al., 2005).

#### 2.2.3 Unitization vs. complex associations in the impact of emotion on memory

Another potential explanation for the opposite effects of emotion on episodic memory (Chiu et al., 2013) can be linked to the dissociation between memory for isolated items vs. memory for relations among items (associative or relational or memory) (Cohen and Eichenbaum, 1993; Cohen et al., 1999; Eichenbaum and Cohen, 2001). There is growing evidence from both animal and human memory research that various memory-related MTL regions can play differential roles in memory for item vs. associations (e.g., memory for an object and memory for the association between the object and its color, size, or context). Specifically, whereas the perirhinal cortex is important for encoding individual items or objects from an experience, the hippocampus (HC) is important for binding distinct item representations into memory (Brown and Aggleton, 2001; Davachi et al., 2003; Ranganath et al., 2004; Tubridy and Davachi, 2011). Further evidence also revealed that the perirhinal cortex may also contribute to some simpler forms of associative learning (Staresina and Davachi, 2010), based on unitization (Graf and Schacter, 1989). Moreover, communication between the HC and PFC plays an important part in the formation and retrieval of association-rich (episodic) memories (Moscovitch et al., 2016). Notably, the PFC regions important for association memory are also involved in emotion processing and emotion regulation (Berkers et al., 2016; Shafer and Dolcos, 2012). Therefore, the unitization of information that involves for instance assembling together different aspects of an event into a single representation via complex associations (e.g., between an object and its color) can be disrupted by emotional information, as memory supporting PFC regions can be “hijacked” by their involvement in emotion regulation operations. Importantly, in some instances, memory for isolated items and for unitized items (where different aspects of the same object are linked into a single representation) can be mediated by similar mechanisms. This is unlike the case of memory representations for more complex associations of different components of an event, as well as associations between temporally separate events, which rely heavily on hippocampal mechanisms (Ezzyat and Davachi, 2014).

Considering such possible dissociations in the available evidence, Chiu et al. (2013) has proposed that emotion enhances memory for both separate and unitized items, but it impairs memories involving more complex, HC-dependent, representations. Consistent with this idea, recent evidence points to increased engagement of the AMY and decreased engagement of the hippocampus linked to opposite effects of emotion on memory for items vs. associations, respectively (Bisby et al., 2016). These findings are also consistent with the *emotional binding model*, which posits that item-emotion binding depends on the AMY and is accompanied by slower forgetting, while item-context associations depend on the HC and are prone to more rapid forgetting (Yonelinas and Ritchey, 2015). However, the idea of differential impact of emotion on memory for unitized items vs. complex associations has yet to be tested rigorously. Also, because most of the studies have tended to focus on associations learned in laboratory settings, it remains unclear how emotion influences reactivation of previous memory representations for real-life events, in forming new associations (Sakaki et al., 2014b).

#### 2.2.4 Reconciling opposing effects of emotion on item vs. relational memory

As mentioned above, the effects of emotion on episodic memory are not uniform. There is agreement that emotion enhances memory for individual items, but how it influences memory for the associated contextual details, or relational memory (RM), has been an issue of debate (reviewed in Bogdan et al., 2024). A prominent view suggests that emotion impairs RM (Bisby and Burgess, 2017), but there is also evidence that emotion enhances RM (Dolcos et al., 2017a). To reconcile these diverging results, a recent investigation by Bogdan et al. (2024) performed three studies incorporating the following features: (1) tested RM with increased specificity, distinguishing between subjective (recollection-based) and objective (item-context match) RM accuracy, (2) accounted for emotion-attention interactions via eye-tracking and task manipulation, and (3) used naturalistic stimuli with integrated item-context content. Challenging the view that emotion always impairs RM, this report identified both enhancing and impairing effects. Specifically, emotion enhanced subjective RM, separately and when confirmed by accurate objective RM. Emotion impaired objective RM through an attention capturing effect, but it enhanced RM accuracy when attentional effects were statistically accounted for using eye-tracking data. Third, emotion also enhanced RM when participants were cued to voluntarily focus on contextual details during encoding, likely by increasing item-context binding, as a results of disengaging from the attention-capturing emotional content. Finally, functional MRI data recorded from a subset of participants showed that emotional enhancement of RM was associated with increased activity in the medial temporal lobe (MTL) and the left ventrolateral prefrontal cortex (vlPFC), along with increased intra-MTL and vlPFC-MTL functional connectivity (Bogdan et al., 2024).

Interestingly, contrary to the view that emotion impairs memory for contextual details by inhibiting recollection-processing brain regions (HC) (Bisby and Burgess, 2017), this study found evidence of synergistic involvement of MTL regions involved in emotion (AMY) and recollection (HC) processing associated with enhanced RM by emotion. Moreover, the fMRI results point to MTL and PFC mechanisms consistent with a model of dual enhancement of associative memory by emotion (the DEAME model, Figure 6), linked to the MTL engagement orchestrated by left vlPFC influences. Specifically, maximized enhancement of subjective confirmed by objective RM when focusing on emotional aspects of stimuli was predicted by the engagement of an *emotion-to-memory* MTL route, reflected in increased activation of the AMY and HC along with functional coupling between these regions. In contrast, maximized enhancement of objective RM (item-context binding) when focusing on the contextual details of emotional stimuli was predicted by the engagement of a purported *perception-to-memory* MTL route reflected in heightened HC activation and connectivity with the PPA. Importantly, both routes are susceptible to top-down modulation from a left vlPFC area (Bogdan, et al., 2024).

These findings disrupt the status quo and have important practical applications. Affective disorders, such as depression and anxiety, along with PTSD are associated with maladaptive memory processing, resulting in memory *de*contextualization Enhanced RM by emotion through voluntary attentional focus points to possible evidence-based solutions on how these patients could grapple with unwanted emotional troubles via redirecting their attention. It is also worth noting that, outside of affective disorders, some of the strongest declines in memory, such as those associated with aging, are linked to RM. Hence, our findings also inform potential attention-based techniques that can be taught to help older adults counteract memory declines. Finally, this research also points to the role of attention in focusing on positive aspects of our experience (Denkova et al., 2015), not just away from negative ones, to increase memory and well-being.

Overall, opposing effects of emotion on episodic memory^6^ have been identified in terms of dichotomies involving three main dissociations: central vs. peripheral, high vs. low priority, and item vs. associations. Future research should consider such dissociations, to further delineate the impact of emotion on memory and the associated neural correlates, according to the type of associations, and linked to effects of emotional stimuli vs. emotional states and to modulations by previous memory representations (Sakaki et al., 2014b). This section also discussed novel evidence reconciling opposing effects of emotion on RM, which revealed fMRI findings consistent with a DEAME model of dual enhancement of associative memory by emotion in the MTL. Finally, because voluntary focus on contextual details during encoding reduces the typical attentional bias (and the associated experienced emotions; Dolcos et al., 2020a, Dolcos et al., 2020b) and enhances associative memory (Dolcos et al., 2020a; Dolcos et al., 2020b; Bogdan et al., 2024), the findings discussed here also point to possible attention-based training interventions to increase RM specificity in healthy functioning, PTSD, and aging, by promoting item-context binding and diminishing memory *de*contextualization.

It should be noted that congruent effects of emotion (e.g., enhancing) at different neurocognitive levels likely involve overlapping processes. For instance, prioritization of processing emotional information at a perceptual level leading to enhanced memory for emotional information is associated with overlapping engagement of neurochemical (noradrenergic), cognitive (attentional), and neural (amygdalar) aspects. This is similar to the link discussed in the next section, but there the overlapping mechanisms were identified linked to divergent effects of emotion across different processes (e.g., working vs. episodic memory). Interestingly, in both cases, dissociable mechanisms mediating within- and across-domains opposing effects of emotion were also identified.

## 3 Opposing effects of emotion across cognitive domains

Available evidence also suggests that opposite effects of emotion can be identified when linking *immediate* (impairing) and *long-term* (enhancing) effects of distracting emotional information across different domains. Specifically, there is evidence that task-irrelevant emotional distracters can impair ongoing cognitive processing (e.g., perceptual), while also leading to enhancement of memory for the distracters themselves. As discussed below, brain imaging studies have identified common and dissociable neural mechanisms for these opposing effects of emotional distraction. These studies provide neurobiological support for linking possible opposing effects of emotion in real-life situations. As alluded to earlier, task-irrelevant emotional information (passing the scene of a tragic accident while driving) may temporarily distract us from the main task (driving), while also leading to better memory for the distracting information (increased memory for the totaled cars).

### 3.1 Opposing effects of emotion on perception vs. episodic memory

**Figure 7:**
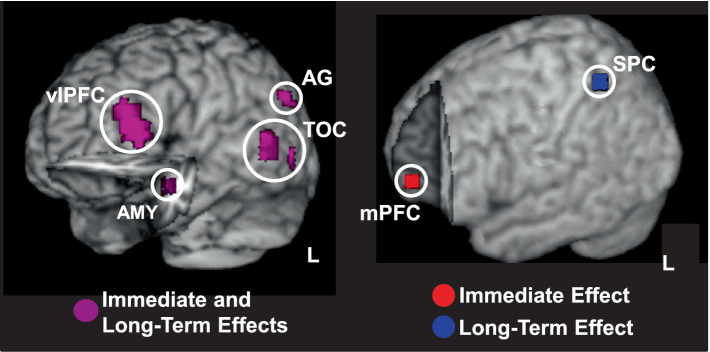
Overlaps and dissociations between brain regions involved in opposing effects of emotion on perception and episodic memory. Left panel shows overlapping responses in cortical brain regions linking the immediate/impairing effect of emotional distraction and the long-term/enhancing effect of emotional memory (shown in magenta); cut-out in the left hemisphere reveals similar responses in the AMY. Right panel shows responses dissociating between the two opposing effects of emotion, with the mPFC involved only in immediate impairment (in red) and SPC involved only in long-term enhancement (in blue). Activation maps are superimposed on high resolution brain images displayed in 3-D views using MRIcron (http://www.mccauslandcenter.sc.edu/mricro/mricron/). AG, angular gyrus; AMY, amygdala; mPFC, medial prefrontal cortex; SPC, superior parietal cortex; TOC, temporo-occipital cortex; vlPFC, ventrolateral PFC; L, Left. Adapted from Shafer and Dolcos (2012), with permission.

Studies examining these effects are still scarce, but available evidence suggests that emotional distraction can, indeed, have an immediate impairing effect on perceptual processing (Shafer et al., 2012), while leading to long-term enhanced memory for the distracters themselves (Shafer and Dolcos, 2012). This study manipulated both the perceptual processing load of the main cognitive task and the emotional charge of the distracting information, and showed differential effects of the two factors on the immediate and long-term effects of emotion. Importantly, this study provided evidence that immediate/impairing and long-term/enhancing effects of emotional distraction are differentially influenced by the availability of processing resources. Specifically, the strongest immediate impairment of emotional distraction occurred when perceptual load was low, and thus more resources were available to process the distracters. However, the strongest enhancement of memory for the emotional distracters occurred when processing resources were least available (high load). Neurally, links between the two opposing effects were observed in both basic emotion processing (AMY) and higher-order processing (e.g., ventrolateral PFC; Figure 7, left panel) regions, showing overlapping effects of emotion on perception and memory. Instead, dissociations were observed mainly in higher order cognitive brain regions, showing involvement only in the immediate impairing (medial PFC) or long-term enhancing (superior parietal cortices, SPC) effects (Figure 7, right panel). Given that the medial PFC is sensitive to emotional stimuli (Keightley et al., 2003; Scheuerecker et al., 2007) and SPC is part of the attentional network (Corbetta and Shulman, 2002), their involvement in the opposing effects can be attributed to increased emotional and goal-relevant processing of the distracters, respectively.

### 3.2 Opposing effects of emotion on working memory vs. episodic memory

Emotional distraction can produce detrimental effects not only in tasks involving lower-level perceptual processing, but also in tasks involving higher-level processing, such as working memory (WM) (Dolcos et al., 2008; Dolcos and McCarthy, 2006; Dolcos et al., 2007). Again, studies linking immediate and long-term impact of emotion on working vs. episodic memory are scarce, but evidence from a study concomitantly investigating these opposing effects within the same participants revealed that emotional distracters presented during the delay interval between memoranda and probes in a WM task had immediate impairing effects on WM performance, while enhancing long-term memory for the distracters (Dolcos et al., 2013). This provides further evidence for the idea that emotional distracters can divert processing resources from the main WM task to processing emotional distracters (Dolcos and McCarthy, 2006), while simultaneously initiating processing that leads to better memory for the distracters themselves (Dolcos et al., 2013).

**Figure 8:**
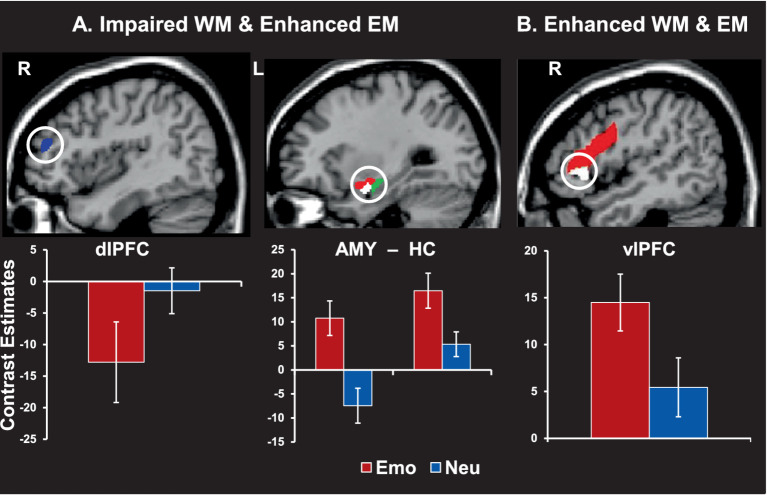
Brain activity linked to working memory (WM) impairment and/or episodic memory (EM) enhancement by emotional distraction. **(A)** Greater deactivation in the dorsolateral prefrontal cortex (dlPFC, blue area, left panel) and increased activity in both amygdala (red area) and hippocampus (green area, middle panel) were linked to impaired WM but enhanced EM performance. **(B)** Increased activity in the ventrolateral prefrontal cortex (vlPFC, red area, right panel) was also linked to enhanced WM and EM. Interestingly, subregions of the AMY and vlPFC (white areas) also had differential contribution to the impact of emotional distraction on WM, with AMY activity predicting impaired WM performance (showing a negative correlation with WM scores) and vlPFC predicting enhanced WM performance (showing a positive correlation with WM scores). The bar graphs show brain activity from peak activation voxels in the highlighted regions. The activation maps are superimposed on high-resolution brain images displayed in sagittal views. R = Right; L = Left; Emo = Emotional distraction; Neu = Neutral distraction. AMY = amygdala; HC = hippocampus. Adapted from Dolcos et al. (2013), with permission.

At the brain level, trials producing both effects (impaired WM and enhanced episodic memory) were associated with decreased activity in dorsolateral PFC (linked to immediate/detrimental impact on WM performance) and increased response in MTL regions (linked to long-term/increased episodic memory performance) (Figure 8A). Of note, the same AMY region was linked to both of these opposing effects (see middle panel). Interestingly, trials associated with enhanced episodic memory performance for emotional distracters that did not disrupt WM performance were linked to increased involvement of top-down PFC mechanisms (i.e., ventrolateral PFC; Figure 8B). This suggests that enhanced memory performance for emotional distracters also benefits from the engagement of coping mechanisms engaged to deal with the presence of emotional distraction during the WM task (Dolcos et al., 2013), possibly involving deeper encoding due to more elaborative processing of the distracters (Dillon et al., 2007).

Overall, these findings demonstrate that the immediate impairing impact of emotional distraction on perception or WM and the long-term enhancing impact of emotion on episodic memory are mediated by overlapping and dissociable neural systems, involving both bottom-up and top-down mechanisms. Interestingly, the link and dissociation between the opposing effects of emotion across cognitive processes/domains could also be seen as downstream consequences of within-domain processing bias, if attention is considered the “domain” of reference. Indeed, the attention-capturing effect of emotion, leading to impaired perception/working memory by task-irrelevant emotional information, can also be responsible for enhanced episodic memory for the distracters themselves. This interpretation is complementary to the view linking the opposing effects of emotion across cognitive processes, leading to impairing immediate vs. enhanced long-term effects, as both have in common an attentional bias in processing emotional distraction.

## 4 Opposing effects of emotion in the stress response

### 4.1 Complex stress-brain interaction orchestrated by neuromodulator action

The impact of emotion on cognition can also be investigated in the context of the response to stressors.^7^ Converging evidence from human and animal studies suggests that the effect of acute stress on cognition, specifically on memory, follows an inverted U-shape function, with moderate levels of stress leading to memory enhancement, and extremes levels of stress (too low or too high) leading to memory impairment (Diamond et al., 2007; Park et al., 2006; Sandi and Pinelo-Nava, 2007). Interestingly, similar effects were also observed in the hippocampal function, in the stress response (Nadel and Jacobs, 1998). Importantly, as also discussed in the next section, highly intense acute emotional events and/or chronic exposure to stressful experiences can create traumatic memories, resulting in long-lasting states of hyperarousal and in the development and persistence of affective disorders (depression, anxiety, PTSD). Indeed, while normal levels of temporary/acute stress can have adaptive function for survival, repeated and prolonged stress can be deleterious for health and survival (McEwen, 1998a, 1998b, 2007). Acute stress can lead to *transient* hyperarousal, which promotes threat detection and memory for emotional events, through the involvement of the AMY and its connections with memory-related brain structures (McGaugh, 2000, 2004), and hence can have an adaptive outcome. By contrast, chronic stress can lead to a state of *continuous* physiological arousal and have deleterious effects on the HC (Roozendaal et al., 2009) and PFC regions (Arnsten, 2000a; Arnsten, 2009; Hains and Arnsten, 2008), hence leading to maladaptive outcomes. It is also important to note that, while historically the exposure and level of stress have been the primary topics of the majority of human and animal neuroscience stress research, recently there has also been an increased effort to understand the role of individual differences in the response to stressors (see Sections 4.2 and 4.3). The main focus here is on acute regulatory processes, as opposed to chronic stress, and the specific effects of acute stressors on cognition are also discussed linked to stress controllability and the role of individual differences (personality, genetic) in stress sensitivity.

Stressful experiences trigger activation of the hypothalamus-pituitary–adrenal (HPA) axis (Joels and Baram, 2009; Lupien et al., 2007), which affects the functioning of both emotion processing brain regions (AMY) (Roozendaal et al., 2009) and regions involved in cognitive processing (HC and PFC) (Lupien et al., 2009; Roozendaal et al., 2009). These three regions are also among the brain areas most sensitive to stress hormones, due to high density of glucocorticoid receptors, and hence not surprisingly they are also the main brain structures involved in emotional learning and memory. Stressors trigger distinct waves of spatially and temporally specific neurochemical changes that affect processing in both affective and cognitive domains (reviewed in Hermans et al., 2014). Initial exposure to stressors is first associated with increased levels of catecholamines (e.g., norepinephrine and dopamine), whose levels get back to normal shortly after stressor offset. Noradrenergic changes are widespread, affecting the whole cerebral cortex, the amygdala, thalamus, and the hypothalamus (Foote and Morrison, 1987), and may have opposite effects on neural functioning in cortical (PFC) vs. subcortical (AMY) regions (Arnsten, 1998; Arnsten, 2000b; Qin et al., 2009; van Marle et al., 2009). Dopaminergic changes occur mostly in the PFC, but also affect responses in the basal ganglia, both in ventral (nucleus accumbens) and in dorsal (caudate nucleus) striatal regions (Abercrombie et al., 1989).

In addition to the fast increases in catecholamine levels, the stress response is also associated with increases in corticosteroid levels. Corticosteroids (cortisol in humans) start to reach the brain after several minutes and when neurons are reached, they exert fast non-genomic and slower genomic effects. At a non-genomic level, corticosteroids interact with membrane-bound mineralocorticoid and glucocorticoid receptors, which are co-expressed in the hypothalamic paraventricular nucleus, AMY, and HC, but the glucocorticoid receptors are predominant in most brain regions, including the PFC (de Kloet et al., 2005). Corticosteroids also interact with catecholamines, increasing norepinephrine levels in AMY (McReynolds et al., 2010), potentiating the effects of stress on dopamine release (Saal et al., 2003), regulating dopaminergic projections within the PFC (Butts et al., 2011), and enhancing AMY function (Roozendaal et al., 2006; Roozendaal et al., 2008). The slow genomic effects are based on transcription modulation affecting levels of multiple proteins that, in turn, affect neuronal function in multiple brain regions, over the course of hours. For instance, genomic effects modulate PFC activity and connectivity (Yuen et al., 2011) and dorsal HC activity (Karst and Joëls, 2005), a few hours after stress induction or corticosteroid application, in a way that they contribute to a normalization in the aftermath of an acute stressful event (Henckens et al., 2010, 2011; Henckens et al., 2012).

Turning to the behavioral consequence of stress responses, there is a large body of evidence from animal and human research showing that stress can have both beneficial and deleterious effects on learning and memory, reflected in enhanced encoding and consolidation of emotional events vs. impairing memory retrieval and working memory, respectively (Lupien et al., 2007; Roozendaal et al., 2009). As mentioned above, while normal levels of temporary/acute stress can have adaptive function for survival, repeated and prolonged stress can be deleterious for health and survival (McEwen, 1998a, 1998b, 2007). Opposing effects in the response to stressors can also be observed in smaller time windows. Evidence points to time-dependent manner of stress influences on brain function, affecting activity and connectivity of visual, emotional, and cognitive processing brain regions in an opposite manner, in order to overall serve adaptation to changing environmental demands. Temporal effects of cortisol on affective and cognitive functions have started being investigated relatively recently (Henckens et al., 2010, 2011; Henckens et al., 2012; Hermans et al., 2014). For instance, Henckens et al. (2012) investigated the time-dependent impact of cortisol on the neural correlates of attentional processing by using a randomized, double-blind, placebo-controlled approach, involving the following 3 groups: placebo, *fast* cortisol, and *slow* cortisol. First, results indicated that the rapid effects of corticosteroids were associated with increased bottom-up/stimulus-driven attentional processing, which caused impaired selective attention (as reflected in increased emotional interference). Neurally, these effects were associated with increased AMY activity and increased AMY-PFC connectivity while processing aversive relative to neutral distraction. These findings from the *fast* cortisol group suggest that the rapid corticosteroid effects cause stimulus-driven behavior, and can contribute, together with those of catecholamines, to a state of hypervigilance (Joels and Baram, 2009; Roozendaal et al., 2006).

Second, the slow effects of corticosteroids modulated the neural correlates of sustained attention, by reducing bottom-up processing. Specifically, the *slow* cortisol group showed reduced activation in visual brain regions linked to sustained attentional processing, as well as reduced negative connectivity between activity in the AMY and insula. These findings suggest that the slow corticosteroid effects might counteract the rapid effects by reducing automatic visual/stimulus-driven processing, and enhance the engagement of more controlled processing, to restore brain functions following stress (Dolcos, 2014). Therefore, this study proposes a more adaptive view on the impact of cortisol on attention and emotion according to the temporal profile of action, with an initial effect optimizing detection of potential threat at the cost of impaired cognitive processing, and a delayed effect normalizing cognitive brain functions following stress (Hermans et al., 2014; Joels and Baram, 2009).

Overall, extant evidence highlighting carefully orchestrated effects on executive control regions such as the PFC, and on limbic structures, such as AMY, suggests that exposure to acute stress increases activity in brain regions involved in fear and attentional vigilance, at the cost of executive control regions’ function. This allocation of resources to the affective vs. executive control function reverses, as the stress subsides, normalizing the emotion-cognition balance in the aftermath of stress (Hermans et al., 2014). Notably, while these effects might allow for optimal responding to stressful situations and subsequent recovery in healthy functioning, they are likely impaired in clinical conditions such as PTSD, which is characterized by a continuous state of hypervigilance (Dolcos, 2013), as discussed in Section 5.

### 4.2 Presence vs. absence of controllability in the stress response

**Figure 9:**
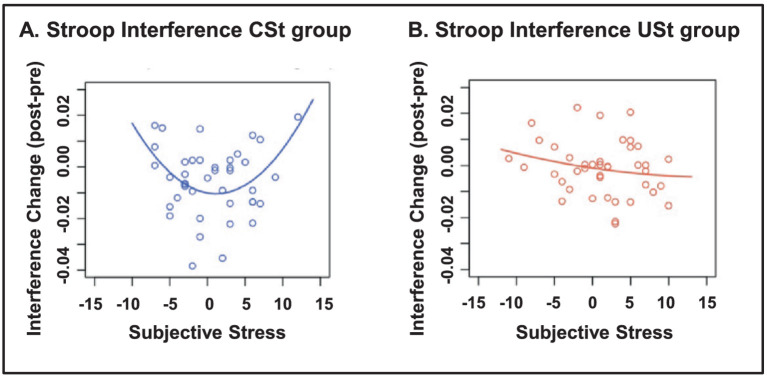
Stroop interference as a function of controllability and subjective stress. **(A)** In the group with controllable stress (CSt), moderate levels of subjective stress were associated with improved Stroop performance (reduced interference), whereas low or high levels of subjective stress were related to impaired Stroop performance (left panel). **(B)** In the group with uncontrollable stress (USt), subjective stress was not related to Stroop performance (right panel). From Henderson et al. (2012), with permission.

Interestingly, the effects of stress on cognition are also influenced by other factors, such as the subjective or objective *controllability*^8^ of the stress. This may explain why in some circumstances and/or individuals stress impairs cognition, whereas in others it may enhance it. There is evidence that the presence of controllability can improve cognitive performance, whereas in uncontrollable situations extremely subjective experience of stress can have detrimental effects on cognitive functioning. The feeling of controllability appears to affect the functioning of the PFC, which inhibits the stress response in the AMY and hence can lead to resilient behavior (Buetti and Lleras, 2012; Henderson et al., 2012; Kerr et al., 2012). For instance, Henderson et al. (2012) investigated the effects of stress controllability and subjective perception of stress on performance on a color-word Stroop task separated by a stress induction block, which was controllable for some participants and uncontrollable for others. Interestingly, controllable stress that was experienced as moderately intense was linked to improved performance (reduced interference), whereas uncontrollable or extreme stress impaired performance (Figure 9). Similarly, even just the subjective feeling of control seems to affect performance (Buetti and Lleras, 2012; Mereu and Lleras, 2013). Buetti and Lleras (2012) investigated the effects of the feeling of control on time perception (estimated duration) of emotional events, and showed that in situations of feeling of control emotion does not impact time perception, whereas in the absence or low feeling of control, time perception is impacted by emotion – that is, negative events are perceived as longer-lasting than positive events, regardless of their level of arousal.

At the neural level, the presence of stress controllability has been associated with the involvement of the ventromedial PFC (vmPFC) (Kerr et al., 2012). Investigation of the effects of stress controllability on the neural correlates of anticipatory response to aversive stimuli in snake-phobic participants showed that controllable anticipatory responses were associated with increased vmPFC activity (Kerr et al., 2012). This finding provides evidence for its involvement in reducing stress responses when stress is controllable, likely by inhibiting AMY responses and promoting resilient behavior. On the other hand, decreased activity in vmPFC has been observed during repeated stressful tasks in subjects who had experienced early-life stress (Wang et al., 2013), which could also be linked to stress uncontrollability in those participants. Specifically, during a repeated stressful task, subjects who had experienced early-life stress, and were also high in trait rumination, had reduced vmPFC activity in the later compared to earlier stressful trials. However, subjects who had experienced early-life stress, but were high in trait mindfulness showed sustained vmPFC activity, and subjects without history of early-life stress had an increased vmPFC response over time. Together, these findings suggest that the presence of control (or the feeling thereof) during stressful situations engages PFC mechanisms that regulate emotional reactions in AMY, and the engagement of these mechanism is affected by previous stress history and personality traits.

### 4.3 The role of individual differences in the stress response

Although research showed that stress can have important consequences on cognition and behavior, and hence can impact physical and psychological well-being, it is also known that stress is not experienced the same way by different individuals (Johnstone and Feeney, 2015). Here, we briefly discuss personality-related differences and then highlight evidence regarding genetic differences in the stress response. Regarding personality differences, neuroticism has been probably the most studied trait regarding individual differences in the response to stressful situations (Canli, 2004; Everaerd et al., 2015; Gunthert et al., 1999), and there is also evidence that high neuroticism is also linked to clinical conditions, such as anxiety and depression (Ormel et al., 2013). As discussed above, there is evidence pointing to individual differences in personality traits indexing coping mechanisms (Wang et al., 2013), and their link with brain functioning associated with early-life stress exposure. The differential vmPFC activity in individuals who experienced early-life stress and were high in trait rumination *vs.* high in trait mindfulness, highlight the response of this region as an important neuroimaging marker distinguishing stress vulnerability vs. resilience in individuals with early-life stress. In addition, early-life stress exposure can also interact with individual differences linked to traits reflecting habitual use of emotion regulation strategies, such as reappraisal, which has been linked to better mood and more adaptive stress responses (Khawli et al., 2017).

Hence, consideration of individual variations would help better understand why in the same circumstances, some people may be more susceptible to stress effects and even develop affective disorders, while others are more resistant against aversive effects of stress. Related to this, an important emerging area of research (McEwen, 2016) targets ways of building resilience, particularly in the case of high-demand, high-risk occupations, such as Army service members and first responders (de Terte and Stephens, 2014). Consistent with the evidence mentioned above, one such successful way of achieving this has been through mindfulness training (Jha et al., 2010). Indeed, mindfulness training in soldiers has been associated with benefits to both cognitive and affective functioning (Jha et al., 2015; Jha et al., 2010). Moreover, increased mindfulness has also been linked to increased resilience and less burnout in first responders (Kaplan et al., 2017). Other training programs targeting emotion control strategies have also proven successful in increasing resilience and well-being (Dolcos et al., 2021).

**Figure 10:**
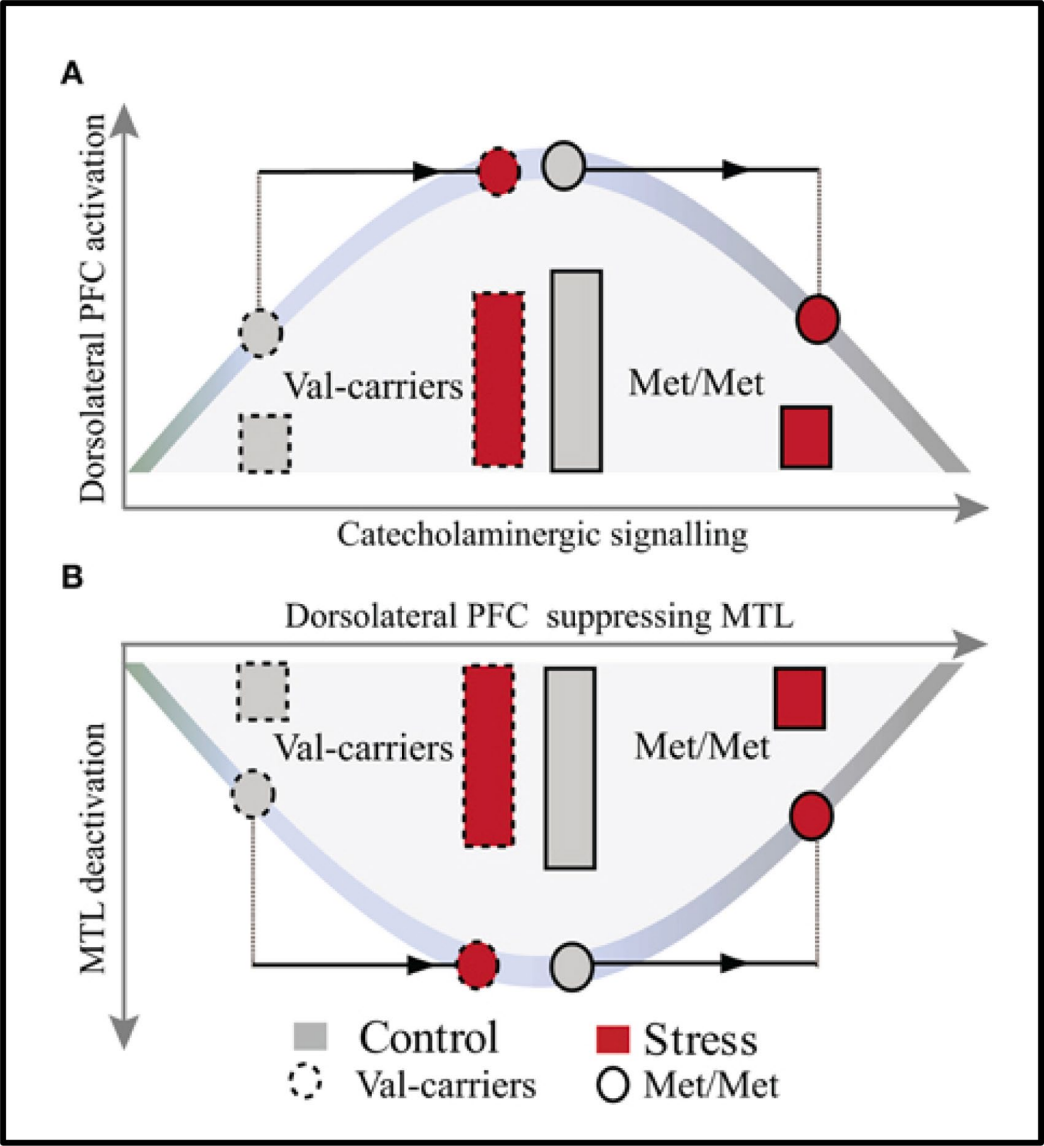
Model illustrating the effects of COMT genotype and moderate stress on WM-Related activations in dorsolateral PFC **(A)** and Deactivations in MTL **(B)**. The model reveals opposite patterns in the effects of moderate stress on dorsolateral PFC activation and MTL deactivation, in Val carriers and Met homozygotes (Met/Met), linked to the basal level of catecholamines. In Val carriers, characterized by suboptimal baseline catecholaminergic activity (see left side of the curve in A, grey-dashed line pattern), moderate stress has positive effects by increasing catecholaminergic activity (top of the curve in A, red-dashed line pattern). This leads to optimal activity in PFC and stronger deactivation in MTL (bottom of the curve in B, red-dashed line patterns). By contrast, in Met homozygotes, characterized by already higher level baseline catecholaminergic activity (top of the curve in A, grey-full line pattern), moderate stress leads to even stronger elevation of catecholamines (right side of the curve in A, red-full line pattern). This results in altered functioning of PFC and less deactivation in MTL (right side of the curve in B, red-full line pattern). PFC, prefrontal cortex; MTL, medial temporal lobe. From Qin et al. (2012), with permission.

In addition to individual variations linked to personality, subjective perception, or previous history of stress, genetic variations can also modulate the effects of stress. Evidence suggests that the opposing effects of stress on memory could be linked to variations in the gene encoding Catechol-O-methyltransferase (COMT), which are linked to individual differences in basal catecholaminergic availability (Qin et al., 2012). For instance, Qin et al. (2012) investigated how COMT genotype (COMT Met homozygotes vs. Val carriers) modulates the effects of moderate stress on WM performance and the associated neural correlates. Behavioral and fMRI data were recorded while participants performed an N-back WM task preceded and followed by either stressful or neutral movies. The results revealed COMT genotype-dependent effects of stress on WM performance and on WM-related activations, in the PFC, and deactivations in the MTL. Specifically, moderate stress led to negative impact in COMT Met homozygotes (characterized by higher baseline catecholaminergic activity), and positive impact in Val carriers (Figure 10).

These effects appear to depend on the baseline catecholaminergic activity, and to follow an inverted-U curve. Val carriers start with a lower (sub-optimal) baseline level of catecholaminergic activity and under moderate stress this activity increases to optimal levels so that it leads to optimal performance and increased dorsolateral PFC activation together with stronger deactivation of the MTL, extending to the AMY. The opposite pattern is observed in Met homozygotes, which start already with higher baseline levels of catecholamines, and moderate stress induction leads therefore to their stronger elevation resulting in impaired performance and decreased PFC activation together with less deactivation in the MTL. Overall, these findings suggest that COMT Met-homozygotes are more susceptible to detrimental effects of stress, whereas Val-carriers are more resilient.

Collectively, evidence regarding the impact of stress on cognition suggests opposite effects linked to the level of stress, the presence or absence of controllability, and linked to individual differences in personality traits and genes. Namely, optimal and controllable levels of stress can have beneficial effects on cognition and behavior, whereas extreme and repeated stress impairs cognition and may lead to the development of affective disturbances. Neurally, the available evidence suggests that the actual/objective presence, or just the mere subjective feeling, of control over stressful situations engages PFC mechanisms that regulate emotional reactions in the AMY. Moreover, PFC functioning in response to stressors has been also linked to individual variations in personality traits indexing vulnerability to (trait rumination) or resistance against (train mindfulness) emotional dysregulation, as well as to genetic differences associated with susceptibility to (COMT Met-homozygotes) or resilience against (Val-carriers) stress. This sections also points to training interventions to increase resilience and well-being (Dolcos et al., 2021; Jha et al., 2010).

## 5 Linking opposing effects of emotion on cognition in affective dysfunctions: the case of PTSD

**Figure 11:**
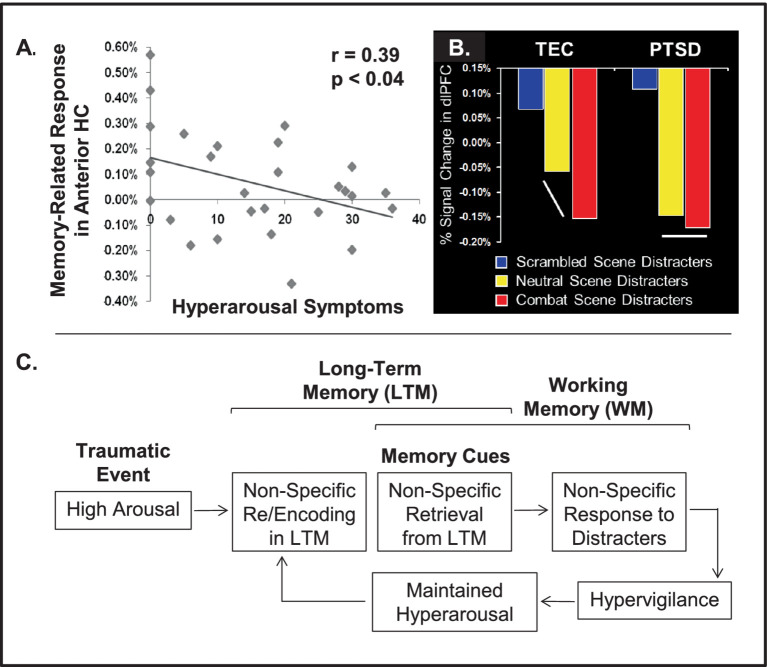
Changes in MTL and prefrontal regions activity pointing to a possible link between enhancing and impairing effects of emotion, in PTSD. **(A)** Reduced memory-related activity for trauma-related pictures in the anterior hippocampus (HC) linked to increased symptoms of arousal suggests impaired encoding of traumatic memories. Adapted from Hayes et al. (2011), with permission. **(B)** Comparison of brain activity in dlPFC during the active maintenance period of a working memory task in the PTSD and Trauma-Exposed Control (TEC) groups points to non-specific disruption of dlPFC response to salient task-irrelevant distracter scenes in the PTSD group, which unlike the TEC groups showed an undifferentiated response in the dlPFC to combat and neutral distracters. dlPFC = dorsolateral prefrontal cortex. Adapted from Morey et al. (2009), with permission. **(C)** Diagram illustrating a possible link between the impact of emotion on long-term memory and working memory in PTSD, which could be initiated and maintained due to non-specific effects of heightened arousal. From Dolcos (2013), with permission.

In vulnerable individuals, stressful life events may cause PTSD, which is associated with highly intense and intrusive memories and thoughts that disrupt normal daily functioning. This clinical condition is characterized by changes in both emotional and cognitive processing, typically reflected in increased emotional reactivity (*hypervigilance* toward potential threats in the environment) and uncontrollable recollection of traumatic memories, which reflect impaired cognitive/executive control (Brown and Morey, 2012; Hayes et al., 2012; Rauch et al., 2006; Shin and Liberzon, 2009). These changes are reflected in regions associated with functions that may be enhanced (AMY) or impaired (PFC) by emotion. Findings from a fMRI study of emotional memory showed reduced activity in the AMY-MTL memory system, during memory encoding, suggesting dysfunction of the mechanisms typically involved in emotional memory. Interestingly, this altered brain activity during encoding was accompanied by increased false alarm rates during retrieval, in PTSD participants compared to a trauma exposed control participants (Hayes et al., 2011). This is consistent with non-specific (gist-based) memory for trauma-related material in PTSD, likely due to dysfunctional engagement of the MTL mechanisms during encoding, due to hyperarousal (Figure 11A). In addition, findings from a fMRI study of WM with emotional distraction showed that the PTSD group also had greater trauma-specific activation than the control group in main emotion processing brain regions, including the AMY and vlPFC, as well as in perceptual brain regions susceptible to emotion modulation (e.g., fusiform gyrus) (Morey et al., 2009). Importantly, though, the PTSD group also showed greater non-specific disruption of activity to both combat-related and neutral task-irrelevant distracters in brain regions that subserve the ability to maintain focus on goal-relevant information, including the dlPFC (Figure 11B). This undifferentiated dlPFC response to combat and non-combat distracters in PTSD is consistent with the *hypervigilance* hypothesis that may explain enhanced response to, and distracting effect of, neutral stimuli.

This evidence suggests a link between the initial impact of emotion influencing episodic memory and the impact of their retrieval triggered by trauma-related pictures presented as task-irrelevant distracters during the WM task (Figure 11C). Specifically, reduced AMY-HC engagement during the formation of memory for trauma-related pictures in the episodic memory study may be explained by initial non-specific encoding of gist-based, decontextualized representations, instead of specific and detailed contextual details of the trauma-related memories, due to hyperarousal. This, in turn, leads to non-specific responses in dlPFC, when trauma-related and neutral stimuli (external or internal; Dolcos and McCarthy, 2006; Iordan et al., 2019) are presented as task-irrelevant distracters, and to symptoms of *hypervigilance*, which contribute to the maintenance of a *hyperarousal* state and to non-specific (re)encoding of traumatic memories, in a continuous vicious cycle (Dolcos, 2013). This view is consistent with possible interpretation of PTSD symptoms (e.g., enhanced threat detection, disrupted executive control) as context dependent outcomes linked to a common cause — i.e., over-prioritization of threat-related external stimuli and internal trauma-related thoughts that can disrupt cognitive processing (e.g., Hirsch and Mathews, 2012).

In summary, this evidence points to general and specific emotional and cognitive disturbances in PTSD, which are linked to alterations in the neural circuitry underlying emotion-cognition interactions, and impact both immediate and long-term effects of emotion on working and episodic memory, respectively.

## 6 Comparing opposing effects of emotion on cognition across fields: healthy aging vs. depression

Evidence for opposing influences on emotion can also be identified in comparisons across groups with opposing emotional biases, such as healthy aging (showing a positive affective bias) vs. depression (showing a negative affective bias). Direct comparisons of the neural mechanisms underlying such opposing affective biases could help determine whether the biases observed behaviorally are also reflected in the neural responses associated with differences in the ability to control emotions observed in these two groups (i.e., impaired in depression vs. enhanced in healthy aging). Aging is associated not only with well-known co-morbidities and losses but also with relatively high levels of emotional well-being, possibly as a result of a *positive affective bias* in processing emotional information (Mather, 2012; Mather and Carstensen, 2005). The idea of a positivity bias in aging is supported by evidence showing that older adults tend to (i) pay attention to and remember more positive information (Charles et al., 2003; Isaacowitz et al., 2006; Mather and Carstensen, 2003) and (ii) show reduced processing of negative information, compared to young adults (Grühn et al., 2007; Wood and Kisley, 2006). From a clinical perspective, older adults have lower rates of depression and anxiety disorders compared with younger adults, indicating cohort differences that may reflect an aging-related decrease in negative affect (Jorm, 2000; Kryla-Lighthall and Mather, 2009). However, older adults’ ability to shield their thoughts and emotions from negative situations suggests an enhanced ability to control emotions (Dolcos et al., 2014; Gross et al., 1997). Notably, similar to the contrast discussed in the case of PTSD, the opposing affective biases observed in healthy aging vs. depression could also be seen as potentially reflecting context dependent outcomes linked to a common process — i.e., opposing valence-dependent prioritization in processing affective information.

**Figure 12:**
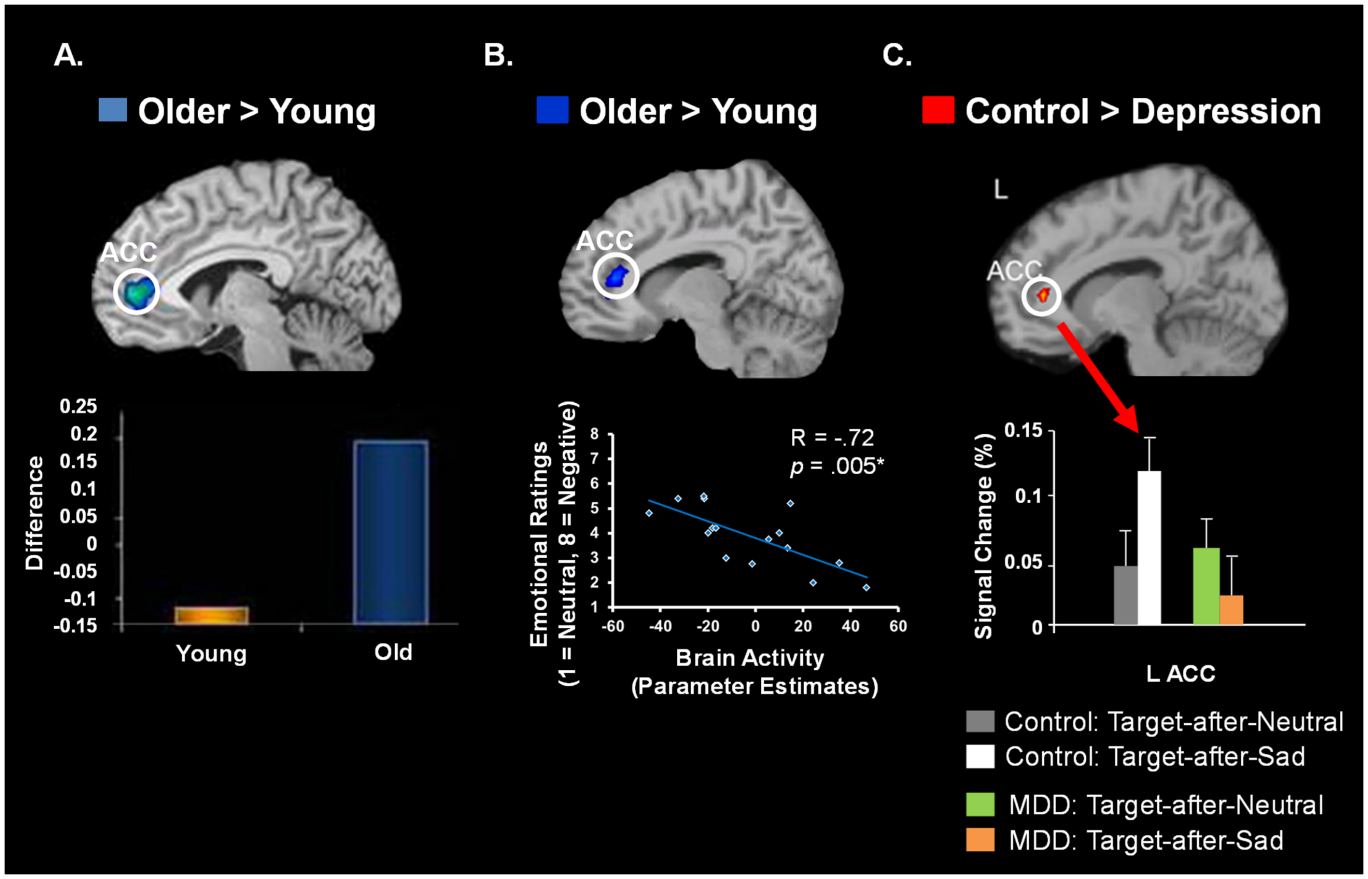
Converging evidence from healthy aging and depression regarding the role of ACC in emotion regulation. Comparison of findings from populations showing contrasting affective biases in the domain of emotion regulation point to similar neural circuitry linked to enhanced (healthy aging; A & B) vs. diminished (depression; **C**) cognitive control of emotion. **(A)** Increased functional connectivity between the amygdala and the anterior cingulate cortex (ACC) in the healthy aging group, who also showed reduced emotional ratings in response to viewing negative pictures. The y-axis represents the difference in trial-level correlations between negative and neutral conditions; adapted from St Jacques et al. (2010), with permission. **(B)** Increased activity in ACC linked to reduced ratings for negative pictures in healthy older adults, consistent with habitual engagement of emotion regulation strategies in this group; adapted from Dolcos et al. (2014), with permission. **(C)** Decreased ACC activity in patients diagnosed with major depressive disorder (MDD), who also showed impaired ability to disengage from processing mood-congruent sad distracters; adapted from Wang et al. (2008), with permission.

Neuroimaging evidence confirms the emotion-regulation account for the positivity bias (for a review, see Nashiro et al., 2012), and points to the role of the medial PFC (mPFC) and the adjacent anterior cingulate cortex (ACC) in the regulation of negative and positive emotions (Dolcos et al., 2014; Gutchess et al., 2007; Kensinger and Schacter, 2008; Leclerc and Kensinger, 2008; St Jacques et al., 2010; Tessitore et al., 2005). Moreover, there is also evidence of increased functional connectivity between the ACC and AMY in healthy older adults, who also showed overall reduced emotional ratings in response to viewing negative pictures (St Jacques et al., 2010) (Figure 12A).

Interestingly, the age differences in the ACC-AMY interactions were associated with changes in the perceived emotional content of negative pictures, reflected in more “neutral” ratings given by the older participants to the negative pictures (St Jacques et al., 2010). This suggests a role of this region in down-regulating the response to negative stimuli, possibly by reducing AMY activity when regulation is successful. This idea was recently confirmed by evidence that activity in similar ACC areas was negatively correlated with the behavioral ratings for negative stimuli in older adults (Dolcos et al., 2014) (Figure 12B), thus providing further support for a role of this region in spontaneous down-regulation of negative emotions in healthy aging.

Interestingly, available evidence links similar brain regions to diminished ability to disengage from processing task-irrelevant negative emotional information in depression (Wang et al., 2008), which is associated with in a negative *affective bias* in processing emotional information (Gotlib et al., 2005; Koster et al., 2005; Siegle et al., 2002). Clinical studies have linked impaired executive control and enhanced emotional distractibility observed in depression to dysfunctional interactions between neural systems involved in cognitive/executive and emotion processing (Drevets, 2001; Mayberg, 1997; Mayberg et al., 1999). Studies using fMRI in depressed patients have reported activity changes in the medial frontal regions (Price and Drevets, 2009), particularly the ACC, as well as in the AMY and other limbic structures (see review by Drevets, 2001). Several studies have also described transient exaggerated activity in ventral frontal and limbic regions, including the AMY (Fu et al., 2004; Sheline et al., 2001; Siegle et al., 2002), in young adults with depression during processing of negative emotional stimuli, although this enhanced activity is not always found (Davidson et al., 2003). Similarly, transient increases or decreases in the dlPFC activity have been reported in depressed patients while performing cognitive tasks (Harvey et al., 2005; Matsuo et al., 2007; Siegle et al., 2007; Wagner et al., 2006). Interestingly, consistent with reduced emotion control in depression, recent evidence (Wang et al., 2008) points to dysfunctional ACC responses following emotional distraction, in areas overlapping with those identified in healthy aging as showing increased response linked to enhanced ability to control emotions (Figure 12C).

The role of these medial frontal regions in the depressive symptomatology is further emphasized by the results of therapeutic interventions showing normalizations of mPFC and AMY activity following pharmacological and non-pharmacological treatments. For instance, pharmacological antidepressant treatments typically report normalization of pre-treatment activity differences in mPFC (Fitzgerald et al., 2008; Mayberg et al., 1999) and AMY (Anand et al., 2007; Sheline et al., 2001). Notably, similar effects in restoring brain function have also been obtained using non-drug therapy, such as cognitive behavioral therapy. Namely, there is evidence of normalized mPFC and AMY activity, following cognitive behavioral therapy, and of a link between increased mPFC activity at baseline and treatment-related improvements (Ritchey et al., 2011). Again, the medial frontal area sensitive to these effects is located in close vicinity to the ACC areas linked to dissociable ability to control emotion in healthy aging (enhanced) vs. depression (impaired) illustrated in Figure 12. Consistent with mPFC/ACC activity as a possible neural marker of treatment-related improvement, there is also evidence of normalized resting-state functional connectivity between mPFC and AMY, consistent with reduced bottom-up influences from emotion processing regions, following emotion control training (Dolcos et al., 2021).

Together, this evidence from groups with opposing emotional biases identify activity in the mPFC/ACC as a biological marker of emotional resilience vs. vulnerability in healthy aging vs. depression, respectively, hence linking its response to differential ability to control emotional responses in these groups. Importantly, therapeutic interventions improve emotion regulation processes in depressed patients by normalizing activity in these areas. Therefore, direct comparisons of these groups with opposing emotional biases and emotion regulation abilities provide an exciting research avenue in addressing mental health issues associated by emotional dysregulation. Such studies can lead to identification of additional neural markers that can be targeted in therapeutic interventions.

## 7 Conclusions and future directions

The overarching goal of the present review was to discuss emerging findings from studies identifying enhancing and impairing effects of emotion on cognition at different levels of analysis. Available research provides evidence that these opposing effects of emotions can be observed within *the same cognitive domains*, *across cognitive domains*, at the more general level of the *response to stressors*, as well as *within clinical groups* and *across groups* with opposing affective biases. Importantly, these multilevel^9^ relations are also influenced by individual differences, which underlines the need for adopting a comprehensive view in studies examining emotion-cognition interactions, in both healthy and clinical populations. The main conclusions of the present review are summarized below and followed by open questions for future research.

Investigation of the opposing effects of emotion within the *same cognitive domains* focused mainly on enhancing and impairing influences on perceptual/attentional and episodic memory processes. Findings concerning the impact of emotion on visual perception and attention point to the critical role of intrinsic factors, such as prioritization of emotional information processing and differential susceptibility to modulation by attention along the time-course of the emotional response, in eliciting enhancing and impairing effects. In addition, task-related contingencies, such as the context of the emotional information (task-relevant vs. irrelevant) and its presentation timing relative to non-emotional information (simultaneous vs. asynchronous), also play a substantial role in these effects. Neurally, these opposing behavioral effects are predominantly linked to the involvement of the AMY, which is sensitive to initial bottom-up prioritization and influences allocation of cortical resources to process emotional information. Similarly, the opposing effects of emotion on episodic memory have been linked to dissociable engagement of the AMY according to the differential impact of arousal on various aspects of the information to be remembered (e.g., central vs. peripheral, high- vs. low-prioritized). More generic dissociations between singular or unitized items’ encoding and formation of complex associations, also contribute to the opposing effects of emotion on different aspects of episodic memory. In this more comprehensive perspective, emotion may lead to memory enhancement of separate as well as unitized items, but to impairment of more complex HC-dependent memory representations. However, novel evidence provides reconciling evidence regarding the impact of emotion on RM, which highlights the importance of considering different aspects of emotional events and their complex interactions that lead to successful memory formation and retrieval.

Evidence from studies investigating impairing and enhancing effects of emotion *across cognitive domains*, such as perception and WM vs. episodic memory, points to both overlapping and dissociating mechanisms involved in the two opposing effects. Bottom-up AMY-MTL mechanisms are involved in both the impairing and enhancing effects of emotion on perception/WM vs. episodic memory. Top-down PFC mechanisms dissociate between the enhancing and impairing effects, pointing to a dorsal-ventral distinction between PFC mechanisms involved in maintenance of goal-relevant information (dlPFC), and the ones involved in coping with emotional distraction linked to enhanced episodic memory for the distracters themselves (vlPFC).

The opposing effects of *acute stress* on cognition have been linked to a variety of factors, ranging from the objective properties of the stressors and the subjective experience of stress to individual variations in personality traits and genotype, reflected in a differential engagement and interplay between MTL and PFC mechanisms. Available evidence points to carefully orchestrated neuromodulatory effects on executive control regions such as the PFC, and the limbic and subcortical structures such as the AMY, involved in emotional and attentional vigilance. Initial involvement of the latter comes at the cost of the engagement of the former, but as the stress subsides allocation of resources to the affective and executive control function reverses, hence normalizing the emotion-cognition balance in the aftermath of stress.

The opposing effects of emotion tend to co-occur and are both deleterious in affective disorders, such as PTSD, where uncontrolled recollection of distressing memories leading to impaired cognition due to emotional distraction could be linked to non-specific effects of heightened initial and perpetuated arousal. These effects also point to alterations of both bottom-up and top-down mechanisms in affective disorders. Finally, evidence from *across-fields comparisons* of groups with opposing emotional biases, such as healthy aging (showing a positive bias) vs. depression (showing a negative bias), identified the mPFC/ACC as biological markers of emotional resilience/vulnerability. This evidence links enhanced response in this region with increased ability to control emotions, characterizing healthy aging, and decreased response with impaired emotion control characterizing depression. Findings from these groups with opposing emotional biases highlight the benefits of across-group comparisons and suggest that capitalizing on the “elders’ wisdom” in emotion control is a viable strategy in addressing mental health issues.

Despite significant progress in clarifying the mechanisms underlying opposing effects of emotion, important open questions still remain. In the reminder of this section, we will elaborate on some of the most prominent emerging topics that need to be considered in future investigations.

1. Future research on the enhancing and impairing effects of emotion on visual perception and attention could examine how *inter*- and *intra*-individual differences, and/or availability of attentional resource at the time of emotion processing, influence the magnitude of these effects. For example, it would be informative to determine if the relation between individual differences and the degree of emotion’s impact is dependent upon the specific effect examined (i.e., enhancing vs. impairing). There is evidence that enhancing effects (better memory for emotional events) are more stable and systematically observed across individuals, whereas impairing effects (increased emotional distraction) are more susceptible to individual variations (Dolcos et al., 2013). However, it is not known whether individual differences in emotional or cognitive domain (or their interaction) are more predictive of impairing effects, and maybe differentially suited for modulating the link between opposing effects of emotion, linked to various cognitive aspects. That is, it is possible that inter-individual differences in the emotional domain may more optimally explain opposing effects of emotion linked to the attention-insensitive time window of the emotion response, whereas inter-individual differences in the cognitive domain may better explain opposing effects related to time window(s) that are more attention sensitive. Aside from clarifying inter-individual differences, it is also relevant to examine the role of intra-individual/state differences, such as linked to menstrual cycle (Sacher et al., 2013), sleep deprivation (Krause et al., 2017), different developmental stages (Ladouceur, 2012), or recent (traumatic) experiences (Hayes et al., 2012), in the interplay between emotion and cognition. Finally, while the influence of the attentional resources on the impact of emotion on lower level perceptual processes has been clarified (Shafer et al., 2012), it is less clear how manipulation of attentional resources within higher level cognitive processes can modulate the impact of emotion (but see Clarke and Johnstone, 2013) and how they are modulated by intra- and inter-individual differences.
2. Regarding episodic memory, an important issue concerns the opposing effects of emotion on associative or relational memory. The idea of differential impact of emotion on memory for items vs. their associated context has only recently been tested more rigorously (Bogdan et al., 2024). By accounting for attention effects (both with eye-tracking and through task manipulation) and also measuring memory for associations more completely (both subjectively and objectively), Bogdan et al. (2024) demonstrated the circumstances in which emotion impairs or enhances RM. Notably, the latter evidence points to ways in which forgetting the contextual details of intense emotional circumstances or stressful events can be prevented. These findings not only disrupt the status quo at the theoretical level, but also has practical implications about what we can do to control, channel, and capitalize on the emotions’ energy to remember better. Moreover, this study also identified the involvement of specific MTL and vlPFC mechanisms, whose engagement and interaction result in enhanced RM by emotion. The findings reported by Bogdan et al. (2024) are consistent with a model of dual enhancement of associative memory by emotion (DEAME) in the MTL, but more research is needed to identify the contribution of the two MTL routes mentioned above (*emotion-to-memory* and *perception-to-memory*). Other aspects that deserve further attention are related to the clarification of the emotion’s effects on memory for other associations, such as those assessed by tests of source memory (see Ventura-Bort et al., 2024, in the present Research Topic), as well as those involving temporal associations (Bogdan et al., 2023a; Talmi and Palombo, 2024) and spatio-temporal integration.
3. Further insights regarding opposing effects of emotion within and across processes could be provided by linking the interplay between enhancing and impairing effects of emotion with interactions between the main functional networks of the brain. Converging evidence from investigations of *large-scale brain organization* and from affective neuroscience suggests that emotion-cognition interactions elicit specific patterns of response in brain regions associated with the major brain networks. Current models of brain organization (e.g., Bressler and Menon, 2010; Seeley et al., 2007; Yeo et al., 2011) typically describe several major functional networks, such as the central-executive, salience, and default-mode networks, which implement domain-general functions, such as executive control, orienting toward motivationally salient stimuli, and self-referential processing, respectively (for alternative but compatible conceptualizations, see Dosenbach et al., 2006; Dosenbach et al., 2008; Dosenbach et al., 2007; Gordon et al., 2014; Power et al., 2011; Power and Petersen, 2013). Although subtle separations between these networks are still a matter of debate (Gordon et al., 2014; Yeo et al., 2011), evidence suggests substantial overlaps between the *dorsal executive* and *ventral affective* systems identified by investigations of emotion-cognition interactions (Iordan et al., 2013) and the central-executive and salience networks, respectively. The default-mode network has been implicated in various functions linked to emotion, such as retrieval of personally-significant memories and self-regulation (Cabeza and St Jacques, 2007; Denkova et al., 2015; Iordan et al., 2019). In this view, the enhancing and impairing effects of emotion may emerge from synergistic or antagonistic interactions among the large-scale brain networks. Among these networks, the *salience network* appears to most reliably track the emotional response (Lindquist and Barrett, 2012). However, executive aspects of processing involved in the response to emotional distraction (e.g., coping with distraction) seem to involve the cingulo-opercular network, which is anchored in the fronto-insular and anterior cingulate cortices (Gordon et al., 2014). Clarification of these overlaps and dissociations warrants further research.
4. Separation into functional domains subserved by the salience and executive control networks also provides a useful framework for better understanding adaptive changes in behavior associated with the impact of stress on cognition, at different time scales (Hermans et al., 2014). According to this model, in the acute stress phase, up-regulation of the salience network and suppression of the executive control network promotes rapid responses essential for short-time survival, such as fear and vigilance, based on more rigid patterns of behavior, and at the expense of elaborate cognitive control (Hermans et al., 2011). After the stressor subsides, during the recovery phase, a reverse shift occurs which promotes normalization of emotional reactivity and enhancement of higher-order cognition, important for long-term survival (Henckens et al., 2011). Hence, investigating the conditions leading to the recruitment of the salience network in conjunction or in conflict with the other brain networks provides a promising avenue for determining links and dissociations between the opposing effects of emotion and their relevance for psychopathology (Menon, 2011; Sylvester et al., 2012; Uddin, 2014).
5. Furthermore, comparing the brain mechanisms engaged by emotional distraction would potentially allow a more fine-grained dissociation between the network components of the dorsal-executive and ventral-affective neural systems, in the context of active task performance. Although the study of large-scale neural networks (e.g., Dosenbach et al., 2007; Power and Petersen, 2013; Seeley et al., 2007; Yeo et al., 2011) has become possible as a result of assessing resting-state functional connectivity, this method has limited ability to capture dynamic interactions among these networks, and thus it provides only a “static picture” of their connectivity. By contrast, specific task manipulations used by studies of emotion-cognition interactions (reviewed in Dolcos et al., 2011; Iordan et al., 2013) have proven effective in eliciting active dissociations among the major brain networks. Hence, such dual tasks with cognitive/ executive and emotional components provide a useful way of studying active interactions between the large-scale brain networks. For instance, there is evidence suggesting that the salience network mediates the interactions between the fronto-parietal and default-mode networks (Di and Biswal, 2013; Goulden et al., 2014). Biasing toward processing of internal or external information by manipulating the originating source of emotion in the context of dual cognitive-emotional tasks (e.g., Iordan et al., 2019) could provide a direct way of testing this hypothesis. Investigation of these networks could also benefit from the employment of novel approaches designed for enhanced interpretability and effectiveness at relatively limited sample sizes of the typical fMRI studies (Bogdan et al., 2023b).
6. Finally, future investigations of the brain mechanisms involved in the response to and coping with emotional distraction in both healthy and clinical populations would benefit from considering not only distracters coming from the outside world (*external* distraction) but also distracters originating from the internal environment, such as memories or thoughts about distressing events (*internal* distraction). Although previous investigations by us and by others (reviewed in Dolcos et al., 2011; Iordan et al., 2013) provided basic evidence concerning the behavioral and brain mechanisms by which irrelevant emotions interfere with on-going cognitive performance, they focused only on the effects of external distraction, such as emotional pictures, and thus it is unclear whether internal distraction produces similar effects. This issue is important because cognitive interference can be elicited also internally (Smallwood and Schooler, 2006), and is exacerbated in affective disturbances (Dolcos, 2013). Hence, clarifying the role of the internal environment in the impact of emotional distraction on cognitive processing could provide insights into the mechanisms of cognitive interference produced in affective disorders by rumination on distressing memories (Cooney et al., 2010; Nolen-Hoeksema, 1991), which can act as a potent internally-generated emotional distraction (Iordan et al., 2019). Notably, training programs that involve exposure to both internal and external emotional challenges provide the opportunity to strengthen emotion regulation and coping strategies, which can result in increased resilience and well-being (Dolcos et al., 2021). The effectiveness of such training programs could be further increased by complementing them with neuromodulation interventions targeting key brain regions identified by neuroimaging studies (Dolcos et al., 2021; Bogdan et al., 2024).

Overall, the present review emphasizes the need to consider the various factors that can influence opposing effects of emotion on cognition and identifies new avenues for future investigations of emotion-cognition interactions. These issues have relevance for understanding mechanisms of emotion-cognition interactions in healthy functioning and in clinical condition where such opposing effects of emotion tend to be exacerbated and deleterious. The ultimate goal of research in this field is identification of the factors that allow for optimal emotion-cognition interactions, which result in happy, healthy, and productive lives.

## Footnotes

^1^*Enhancing* vs. *impairing* effects of emotion may be associated with *beneficial* vs. *detrimental* effects, respectively, and hence to some extent these notions can be used interchangeably. However, this is not always the case, as dissociations can also be identified. For instance, attending to task-irrelevant emotional distraction may impair performance in cognitive tasks at hand. However, from an evolutionary perspective, having in place neural systems sensitive to emotional information whose detection and processing is relevant for survival (e.g., in threatening situations) is adaptive and hence beneficial. Therefore, the *impairing* effects of emotional distraction may be seen as “necessary side-effects” (or by-products) of *enhancing* effects of emotion on attention and perception, which overall are beneficial for survival. In the case of psychopathology, on the other hand, exacerbation of both enhancing and impairing effects of emotion are context inappropriate and thus indeed detrimental. Specifically, at a basic level, enhanced emotional memory retrieval is beneficial for survival, if it helps us predict/avoid dangerous situations. However, if it causes suffering, such as in the case of PTSD patients, who may inappropriately re-experience memories for traumatic events in actually safe situations, such enhanced memory is maladaptive and hence deleterious. This clarification is important, because a rigid view that impairing effects of emotion (or stress for that matter) on cognition are always detrimental hampers research progress. Hence, in the present discussion, *enhancing*/*impairing* effects refer to the impact of emotion on the cognitive processes or performance measures of interest (perception, memory, etc.), rather than to *beneficial*/*detrimental* effects in general, or from an evolutionary standpoint.

^2^Literature regarding the influence of stress on cognitive processing is discussed in more detail in Section 4.

^3^It is important to note that this section we only discussed research investigating the effects of emotion linked to the characteristics of the stimuli, rather than to the emotional state one may be in. Indeed, one’s emotional state can also impact cognition and behavior, and there is evidence that emotional state can affect visual attention and perception. Perhaps the more widely known phenomenon is the influence of mood on attentional scope, with positive mood linked to increased distributed attention and negative mood to more focused attention (Vanlessen et al., 2016; Whitmer and Gotlib, 2013). Additionally, and perhaps lesser known, is the effect of mood and emotional state on perception. Specifically, loudness, height, and distance judgements have all been shown to be influenced by one’s emotional state (Anderson et al., 2011; Riener et al., 2011; Siegel and Stefanucci, 2011; Stefanucci and Proffitt, 2009; Zadra and Clore, 2011).

^4^The opposing effects of emotion on episodic memory discussed here refer to the characteristics of the information to be remembered (central vs. peripheral; prioritized vs. non-prioritized, items vs. associations), rather than to different memory stages (encoding vs. retrieval). Effects of emotion on different memory stages are discussed in detail elsewhere (Dolcos et al., 2017a; Dolcos et al., 2017b).

^5^Of note, priority can be assigned by bottom-up salience (e.g., emotional) or by top-down (goal-relevant) relevance (Lee et al., 2012; Sakaki et al., 2014a).

^6^Similar to the case of perception and attention, one’s emotional state can also impact episodic memory (Fitzgerald et al., 2011; Greene et al., 2014; Lewis et al., 2005). In short, available research suggests that emotional states can lead to differential effects depending on their congruency with the emotional information to encode and retrieve, a phenomenon known as mood-congruent memory (Blaney, 1986). For instance, memory is enhanced when the mood valence is consistent between encoding and retrieval, or when there is congruency (during encoding or retrieval) between the subject’s mood and the valence of the information to be encoded/retrieved (Fitzgerald et al., 2011). The differential impact of emotional stimuli vs. emotional state is still an open question, as research trying to delineate these effects is scarce (Cohen et al., 2016). Hence, clarification of this issue is a fruitful avenue for future research, both in normal functioning and in affective disorders, which are characterized by an overall negative mood and a negative affective bias in attention, perception, and memory (Drevets, 2001).

^7^Other psychophysiological states, such as being hungry or sleep deprived, may also influence cognitive processing (Benau et al., 2014; Killgore, 2010). However, due to space limitations, in this section we focus only on stress, as a representative emotional state, whose influence on cognition extends beyond the transient effects typically investigated in studies of emotion-cognition interactions (discussed in Sections 2 & 3). See also the associated footnotes from previous sections, pointing to literatures focusing on state-related effects of emotion on perception/attention and episodic memory.

^8^Although some definitions of stress imply uncontrollability, here we refer to studies specifically manipulating controllability.

^9^As discussed, emotional information can enhance or impair cognition depending on various factors, including its relevance to the present goals. For instance, emotion can help perception and memory, when task-relevant, or it can be distracting, when task-irrelevant. In this context, although not the focus of the current review, it is worth mentioning that emotional information also influences the behavioral consequences of affective processing, reflected in motor control/action. Interestingly, recent evidence shows that the valence of emotional facial (Mancini et al., 2022; Mirabella et al., 2023; Montalti and Mirabella, 2023) and body postures (Calbi et al., 2022) expressions elicit a consistent behavioral effect only when they are goal-relevant, regardless of the effectors used to provide responses (e.g., hand, arm, legs) or the type of required responses (i.e., moving or refraining from moving). In contrast, when the stimuli’ emotional content is task-irrelevant, it does not affect motor control; see also Mirabella et al. (2024), for evidence regarding the neural correlates associated with the task-relevant effects mentioned above. Overall, this evidence adds nuance to the present discussion regarding opposing effect of emotion of various aspects of cognitive processing and further points to the importance of also considering the behavioral consequences of emotion processing.
